# An Untargeted Metabolomics Approach to Characterize Short-Term and Long-Term Metabolic Changes after Bariatric Surgery

**DOI:** 10.1371/journal.pone.0161425

**Published:** 2016-09-01

**Authors:** Sophie H. Narath, Selma I. Mautner, Eva Svehlikova, Bernd Schultes, Thomas R. Pieber, Frank M. Sinner, Edgar Gander, Gunnar Libiseller, Michael G. Schimek, Harald Sourij, Christoph Magnes

**Affiliations:** 1 JOANNEUM RESEARCH Forschungsgesellschaft mbH HEALTH Institute for Biomedicine and Health Sciences, Graz, Austria; 2 Medical University of Graz, Department of Internal Medicine, Division of Endocrinology and Diabetology, Graz, Austria; 3 Institute for Medical Informatics, Statistics and Documentation Medical University of Graz, Graz, Austria; 4 eSwiss Medical & Surgical Center, St. Gallen, Switzerland; 5 CBmed – Center of Biomarker Research in Medicine, Stiftingtalstrasse 5, 8010 Graz, Austria; Instituto de Investigacion Sanitaria INCLIVA, SPAIN

## Abstract

Bariatric surgery is currently one of the most effective treatments for obesity and leads to significant weight reduction, improved cardiovascular risk factors and overall survival in treated patients. To date, most studies focused on short-term effects of bariatric surgery on the metabolic profile and found high variation in the individual responses to surgery. The aim of this study was to identify relevant metabolic changes not only shortly after bariatric surgery (Roux-en-Y gastric bypass) but also up to one year after the intervention by using untargeted metabolomics. 132 serum samples taken from 44 patients before surgery, after hospital discharge (1–3 weeks after surgery) and at a 1-year follow-up during a prospective study (NCT01271062) performed at two study centers (Austria and Switzerland). The samples included 24 patients with type 2 diabetes at baseline, thereof 9 with diabetes remission after one year. The samples were analyzed by using liquid chromatography coupled to high resolution mass spectrometry (LC-HRMS, HILIC-QExactive). Raw data was processed with XCMS and drift-corrected through quantile regression based on quality controls. 177 relevant metabolic features were selected through Random Forests and univariate testing and 36 metabolites were identified. Identified metabolites included trimethylamine-*N*-oxide, alanine, phenylalanine and indoxyl-sulfate which are known markers for cardiovascular risk. In addition we found a significant decrease in alanine after one year in the group of patients with diabetes remission relative to non-remission. Our analysis highlights the importance of assessing multiple points in time in subjects undergoing bariatric surgery to enable the identification of biomarkers for treatment response, cardiovascular benefit and diabetes remission. Key-findings include different trend pattern over time for various metabolites and demonstrated that short term changes should not necessarily be used to identify important long term effects of bariatric surgery.

## Introduction

Obesity has become a major health concern over the last decade with approximately 2.3 billion adults currently being overweight (BMI 25–29.9 kg/m^2^) and more than 700 million being obese (BMI ≥30) (WHO)[1]. In addition to direct negative effects of obesity on general health (like hypertension, cardiovascular disease, cancer [2–9]), obesity also significantly increases the risk for co-morbidities such as type 2 diabetes, a condition present in 31% of morbidly obese subjects [10]. Besides type 2 diabetes, obesity is also associated with cardiovascular risk (CVR) factors such as hypertension or dyslipidemia, which considerably increase CVR in obese subjects [2–8].

Bariatric surgery, especially Roux-en-Y gastric bypass, effectively reduces body weight and has also been reported to improve glycemic control in type 2 diabetes patients and to reduce cardiovascular events and overall mortality in obese subjects [2,3,11,12]. Although Roux-en-Y gastric bypass surgery is an effective treatment option for obesity, it is a typically irreversible and costly procedure with potential complications [13,14]. Rapid metabolic improvements within the first few weeks after gastric bypass surgery have recently been described but are difficult to explain by the relatively small weight loss during this short period of time [15,16]. Such rapid metabolic changes and very individual responses to gastric bypass surgery are not yet fully understood [17].

The assessment of individual responses of patients for a more personalized approach requires a detailed understanding of short- and long-term metabolic effects of bariatric surgery. Previous studies have assessed effects of bariatric surgery on metabolic profiles [18–34], but so far mostly short-term effects of bariatric surgery have been studied [34–36], where metabolic changes are influenced by the surgical procedure and the convalescence period. Therefore, short-term changes allow only a limited perspective on long-term effects which are a main factor for an improved sustainable metabolic, cardiovascular and overall outcome. A comprehensive metabolomics profile linking short- and long-term effects is currently missing.

The aim of our study was to investigate both short- and long-term metabolic changes after bariatric surgery and to link metabolic changes to clinical outcomes. We used an untargeted metabolomics approach combined with a data-driven approach for statistical analysis. In addition, we explicitly searched for metabolites known as cardiovascular risk markers.

## Materials and Methods

### Study Population

We used serum samples from a clinical study which had been designed to investigate the restoration of beta-cell function in patients undergoing bariatric surgery. The design and the results of this study have been reported previously [37]. In brief, patients older than 18 years with an indication for bariatric surgery who failed to successfully lose weight with previous lifestyle interventions were included in this trial. Patients with mental incapacity, unwillingness or language barriers precluding adequate understanding or cooperation, pregnant or breastfeeding women or women with the intention of becoming pregnant or not using adequate contraception, patients with severe chronic inflammatory or systemic disease or those with a disease or condition which the investigator or treating physician feels would interfere with the trial or the safety of the subject, were excluded from participating in the trial.

All 44 patients signed an informed consent before they were included in the trial, which was performed at two clinical sites, the Medical University of Graz (AUT) and Interdisciplinary Obesity Center in St. Gallen (CH) (clinicaltrials.gov: NCT01271062). 19 patients were enrolled at the Swiss study-center and 25 patients in Graz. 24 patients had type 2 diabetes mellitus at baseline (11 from Graz, 13 from St. Gallen). Before merging the datasets, a comparison of patients’ characteristics from both centers showed no significant differences in metabolic and anthropometric parameters including BMI, age, HbA1c or blood pressure. Only triglyceride levels at baseline were higher in patients from St. Gallen (mean 197 ±116 mg/dl) than those from Graz (130 ±78 mg/dl), unadjusted p value = 0.04).

The surgical procedure performed in all participating patients was a Roux-en-Y gastric bypass. All patients received dietary counseling (2 times pre-surgery, 7 times post-surgery) according to a standardized protocol recommended by bariatric surgery guidelines at both centers. This also included a protein rich supplementation within the first 4 weeks post-surgery with Resource Protein 88^®^ (Nestle, Switzerland). Further supplementation of vitamins and micronutrients was done according to standardized scheme (S4 Table).

For the current analysis we only included patients with serum samples available at all three different sampling times: two to four weeks before the surgery (PRE), one to three weeks after the surgery (POST) and at a follow-up one year after surgery (FU). Serum samples were taken at both study centers and sample preparation was done according to the same standardized procedures: venous blood samples were collected, blood was allowed to clot (30 min, 25°C), serum was separated by centrifugation (2000 g, 4°C, 30 min) and then immediately stored at -80°C. The samples were shipped on dry ice to Joanneum Research-Health Graz where all subsequent analyses were performed. The study was approved by the local ethics committees (EC St. Gallen und EC of the Medical University of Graz) and conducted in accordance with the principles of the Declaration of Helsinki, GCP-ICH and the requirements of the appropriate regulatory authorities.

### Sample preparation & HPLC-HRMS Analysis

Serum samples were processed as published by Yuan et al [38]. Briefly, 200 μl of serum were transferred to 1.5 ml tubes and centrifuged at 4°C for 10 min at 13,000 g. 800 μl methanol (cooled to -80°C) were added and mixed with the samples. Samples were incubated overnight at -80°C then centrifuged at 13,000 for 10 min and 800μl of supernatant was transferred to 1.5 ml tubes. Samples were evaporated to dryness by using nitrogen and reconstituted in 200 μl 30% methanol in water. Peak splitting was avoided by using small injection volumes (10 μl) for LC-HRMS analysis.

LC-HRMS analyses were performed with an Ultimate 3000 UHPLC system (Thermo Fisher Scientific, San Jose, CA, USA) coupled to a high resolution mass spectrometer Q-Exactive (Thermo Fisher Scientific, Bremen, Germany). The chromatographic separation was done by HILIC (hydrophilic interaction liquid chromatography) on a Luna NH2 column (2×150 mm; 3 μm; Phenomenex, Torrance, USA) following the procedure published by Bajad et al [39]. HILIC retains hydrophilic compounds and is ideal for polar low molecular weight compounds in contrast to the more common reversed phase chromatography [38].

10 μl of each sample were injected to the chromatographic system. Separation was performed using eluent A: 20 mM ammonium acetate + 20 mM ammonium hydroxide in 95:5 water: acetonitrile, pH 9.45; Eluent B: acetonitrile. The gradient was as follows: t = 0 min, 85% B; t = 15 min 0% B; t = 20 min 0%B; t = 22 min 85% B; t = 37 min 85%B. Flow rate was set to 150μl/min. Full scan spectra were recorded in positive and in negative electrospray from m/z 70–1050 with a resolution of 140,000 (at m/z 200).

**Quality Control:** 10 μl of each sample are mixed together to generate a pooled quality control sample (QCs). QCs and solvent blank samples (BLs, 30% methanol in water) were injected sequentially in-between the human serum samples. BLs, each followed by a QC, were measured after every third serum sample, in one single batch, resulting in 46 BL, 46 QCs and 132 serum samples.

#### Identification and annotation of metabolites

Metabolites were identified according to Sumner et al. [40] *(1) Identified compounds* were identified by accurate mass and retention time in comparison to reference standards. Reference solutions were prepared in 30% methanol in water in 1 μg/ml concentrations (*2) Putatively annotated compounds* were annotated by accurate mass comparison using freely available metabolite databases (HMDB, KEGG, Metlin) [41–46].

### Data-Processing

**Fig 1:**
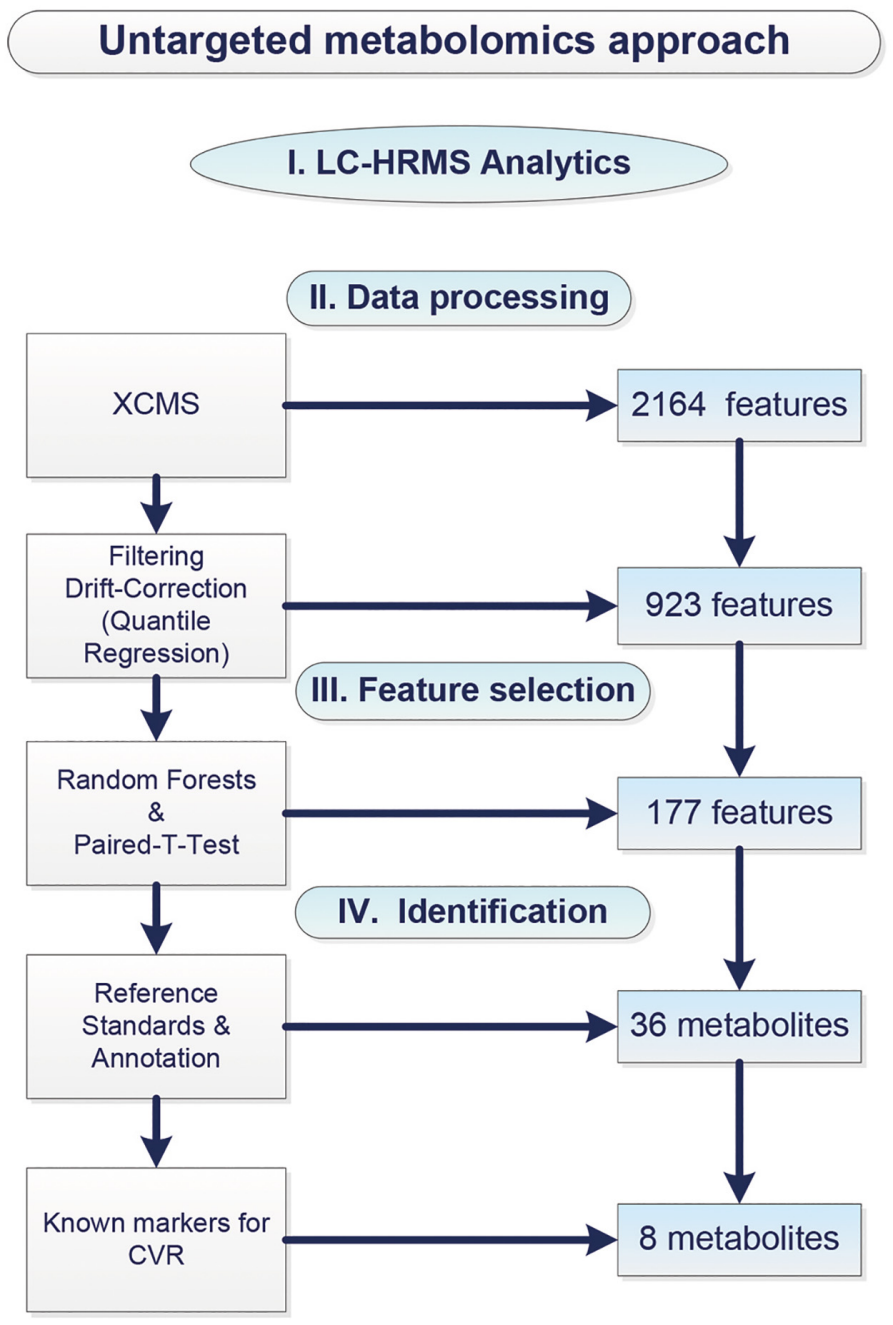
Scheme of untargeted metabolomics approach (CVR = cardiovascular risk), 4 CVD metabolites out of 36 were searched explicitly in the data.

Our untargeted metabolomics approach (Fig 1) was implemented in four steps: (I) LC-HRMS-analysis, (II) data processing, (III) metabolic feature selection and (IV) identification of metabolites.

Raw data was converted into an mzXML file format using ReadW (v4.0.2). The scans from negative and positive electrospray ionization were saved in two different files. Untargeted analysis for both ionization methods was done by using the open source R package XCMS [47,48]. XCMS parameters were optimized by the R-package IPO [49].

The following XCMS-parameters were optimized separately for positive and negative mode: findPeaks.centWave: ppm, min.peakwidth, max.peakwidth, mzdiff; retcor.obiwarp: profStep, gapInit, gapExtend; group.density: bw, mzwid, minfrac.

XCMS-data processing results in a data matrix which contains peak intensities (AUCs) from the negative and positive ionization modes. The peak intensities are called metabolic features, which are a unique combination of retention-time and median m/z ratio.

The order of the serum samples was randomized for analysis to avoid time-dependent bias. To exclude system-peaks (impurities in the measurement-system, visible in blanks) as well as poorly detected metabolic features, filter steps were performed based on QCs- and BLs. A quantile regression approach was applied to correct time-dependent drifts, based on QC-intensities. Therefore, the R-function *quant*.*reg* was applied in two steps to achieve the requested drift correction [50,51]. First based on the QC-intensities a model was built to configure the variation in time. The 50% quantile of the QC’s (y’s) was estimated via nonparametric quantile regression, using regression splines depending on the sample number (x’s). This procedure fitted a piecewise cubic polynomial (breakpoints in the third derivative) with 16 knots (df) arranged at the 50% quantile of the x’s: rq (y ~ bs (x, df = 16), tau = 0.5). Second, another quantile regression model was estimated to attune all samples using a multiplicative correction factor based on the median intensities of the original QC-intensities. After a successful drift correction, metabolic features were used for statistical analysis. A threshold of 30% relative standard deviation of QCs was introduced after drift correction to include only well measured or corrected metabolic features for statistical analysis.

### Statistical Analysis

In addition to our untargeted metabolomics approach (Fig 1), we explicitly searched for significant changes in CVR relevant metabolites such as BCAA which have previously been described to be influenced by bariatric surgery (18,52,53) by applying paired t-tests. All analyses were done in R version 3.1.0 (54). A combination of univariate and multivariate methods was chosen since both selection processes are able to incorporate different information [52].

We applied unsupervised Random Forests (RF) to illustrate clustering and supervised RFs for classification of samples and to consecutively select the most important metabolic features to distinguish between sampling points. The influence of metabolic features on the supervised RF was indicated by Mean-Decrease-Accuracy, which is calculated from the number of correct votes per variable and per node. The higher this value, the greater is the influence of the metabolic feature on the classification. All metabolic features with a Mean-Decrease-Accuracy larger than zero were considered to have an influence on the classification. Supervised RF were performed with 300 trees and 30 variables tried at each split. RFs were done using the R-function *RandomForests* [53,54].

The visualization of the unsupervised and supervised RFs with respect to potential sample clustering was done by using MDS plots (Multi-Dimensional Scaling Plot of Proximity matrix from RFs). MDS plots represent the scaling coordinates of the proximity matrix of RFs.

Paired t-tests were used to detect univariate changes in metabolic profiles between two sampling points (PRE-POST and PRE-FU). P-value-adjustment for multiple testing was done via false discovery rate using Benjamini & Hochberg [55]. An adjusted p-value < 0.01 was considered to be relevant for metabolic feature-selection.

Combining RFs and paired-t-tests defined a process to select metabolic features that describe the changes between two points in time. The same metabolic feature selection process was applied to compare times 1 and 2 (PRE-POST) as well as times 1 and 3 (PRE-FU). The intersection of the selected characteristic metabolic features from both comparisons represents the combined information of short- and long-term effects of bariatric surgery. The success of the metabolic feature selection was visually verified by unsupervised RFs which were used to show clustering of the samples taken before and after surgery.

Trend patterns were analyzed by taking the median intensities of each sampling point from all patients for each selected metabolic feature. We distinguished four different median trend patterns: increasing trends, decreasing trends, V-pattern (decreasing shortly after surgery and increasing after one year) and Ʌ-pattern (increasing shortly after surgery and decreasing after one year).

Data processing detected 2164 metabolic features by XCMS. 923 metabolic features passed the defined quality criteria and were used for further statistical analysis to select relevant characteristic and discriminatory metabolic features. Quantile regression was applied to correct time-dependent drift in the data, indicated by QC-Intensities in measurement order, resulting in an improvement of the median QC-CV from 0.2 to 0.1.

## Results

Serum samples from 44 obese patients included in the study were analyzed (for characteristics of patients see Table 1).

**Table 1:** Clinical characteristics of study population, presented in mean (± SD) for each sampling point: PRE (before surgery), POST (1–2 weeks after surgery), FU (one year after surgery) including p-values from paired t-tests.

	PRE	POST	FU	p-value PRE-POST	p-value PRE-FU	p-value POST-FU
**Gender (male/female)**	15/29	-	-	-	-	-
**Age (years)**	46.8(11.3)	-	-	-	-	-
**Weight (kg)**	126.4(19.5)	117.2(18)	86.3(13.4)	<0.001	<0.001	<0.001
**BMI (kg/m²)**	43.9(5.4)	40.8(5.2)	30(4.4)	<0.001	<0.001	<0.001
**HbA1c (%)**	6.5(1.3)	6.1(1)	5.6(0.8)	<0.001	<0.001	<0.001
**Sys**	132.6(15)	123.7(14.1)	126.4(17.1)	<0.001	0.029	0.338
**Dias**	83.7(10.9)	77.3(9.1)	77.8(11.1)	<0.001	0.003	0.787
**Chol**	181.5(39.7)	-	146(27.9)	-	<0.001	-
**HDL**	50.2(16.7)	-	49.8(14.5)	-	0.8064	-
**LDL**	47.7(53.2)	-	36.8(39.4)	-	<0.001	-
**TG**	159.2(101.3)	-	88.8(32)	-	<0.001	-

**Fig 2:**
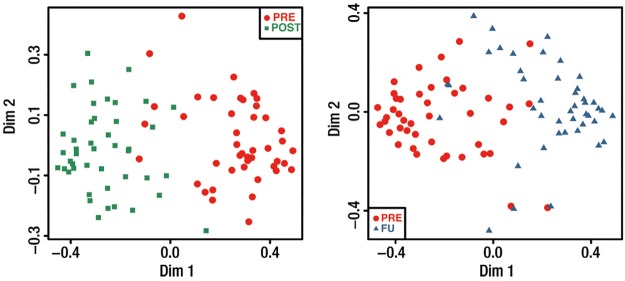
Multidimensional scaling plots from supervised Random Forests of initial 923 metabolic features show distinct clustering of samples taken before (PRE) and after the surgery for both points in time (POST, FU).

**Untargeted metabolic feature selection** resulted in 177 metabolic features that indicate significant short-term and long-term changes, out of which 32 metabolites were successfully identified or putatively annotated (see Table B in S2 Table). A simultaneous explicit search further identified 4 additional metabolites (BCAAs) affected by bariatric surgery. Supervised RFs showed a clear separation between the samples taken before and after surgery, with a class error of only 6% and 11%, respectively (Fig 2).

**Fig 3:**
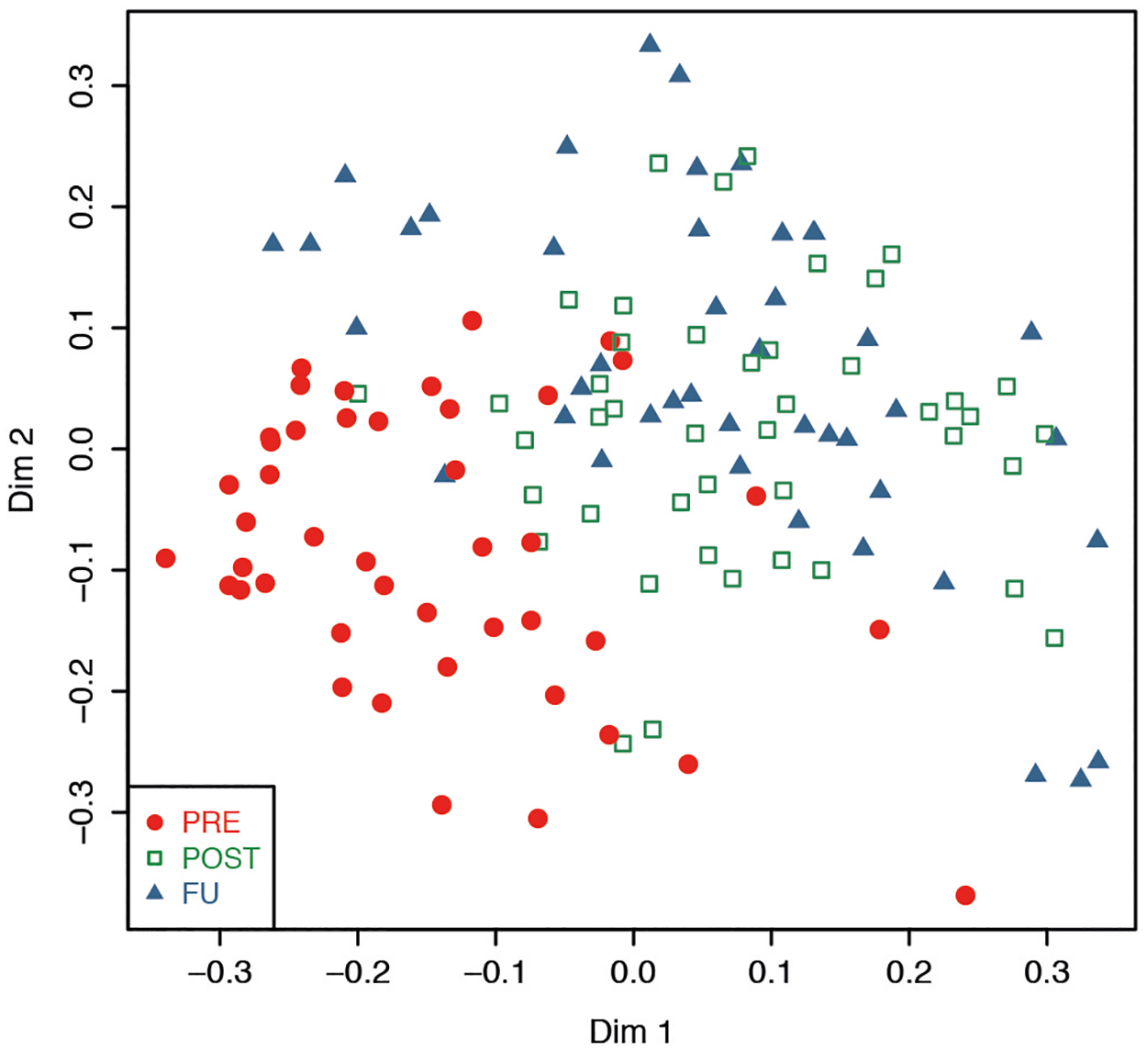
Multidimensional scaling plot of unsupervised Random Forests using 177 selected metabolic features from all three sampling points.

As a visual control, unsupervised RFs were built based on the selected 177 metabolic features. Samples taken before the surgery (PRE) clustered closer together than samples taken after the surgery (POST and FU) (Fig 3).

**Fig 4:**
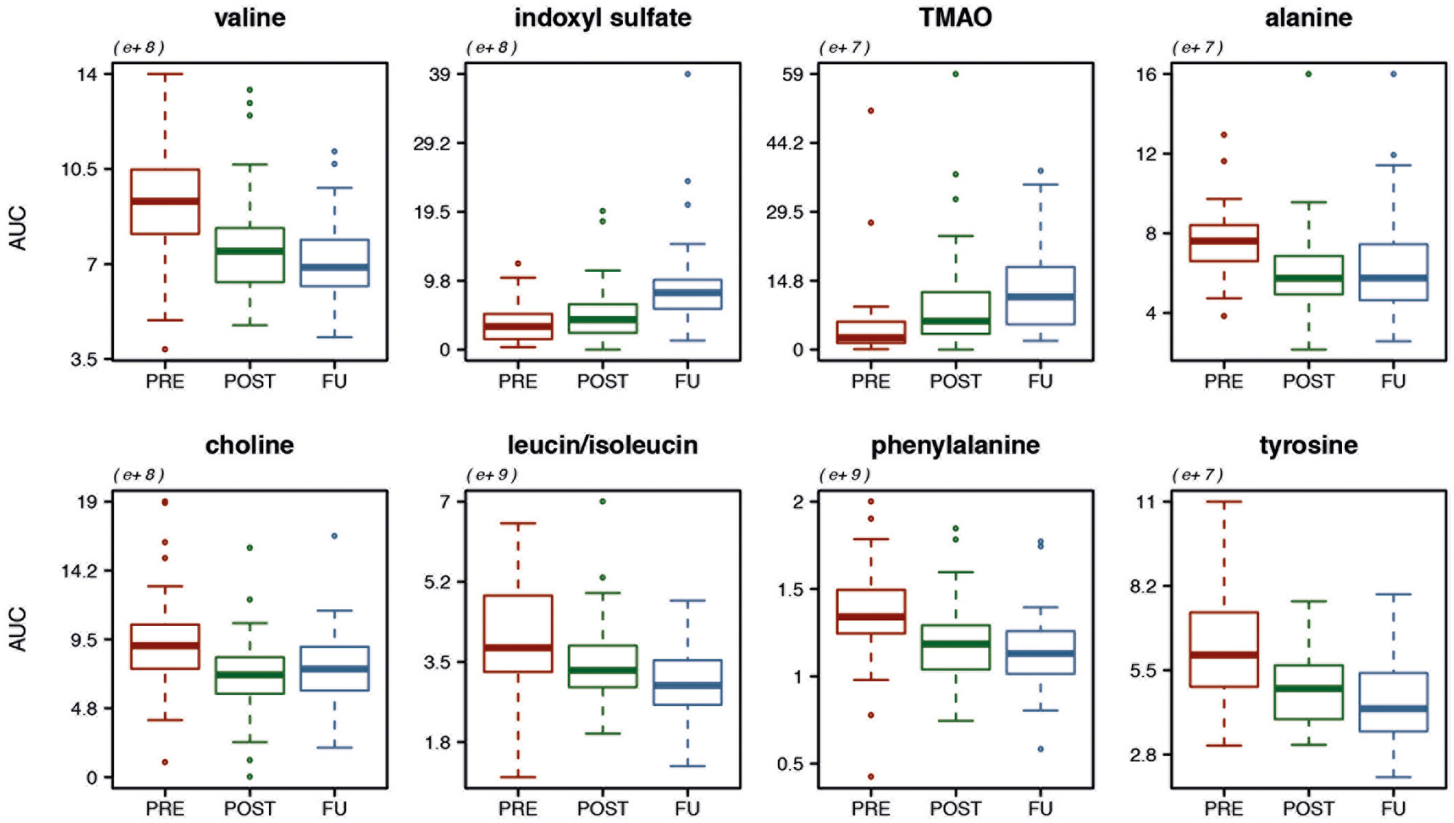
Boxplots of peak-AUC metabolites related to CVR for three different sampling points.

8 identified metabolites were linked to CVR factors [56,57]: TMAO, indoxyl sulphate (increasing trend), choline, alanine, phenylalanine, tyrosine, valine, leucine/isoleucine (decreasing trend) (Fig 4, Table 2).

**Table 2:** Unidirectional trends of changes in the intensities (peak-AUC) of identified metabolites before and after bariatric surgery, metabolites in bold have previously have been associated with CVR.

Metabolite* (Ionization-mode)	MzMed	RtMed	p-value** PRE-POST	p-value** PRE-FU	Ratio*** PRE,POST	Ratio POST, FU	Ratio PRE,FU
		decreasing trend					
**Alanine (+)**	90.0556	12.20	<0.001	0.019	0.8	1	0.85
**Choline (+)**	104.1076	10.06	<0.001	0.003	0.74	1	0.79
**Leucine/Isoleucine° (+)**	132.1022	9.45	0.003	<0.001	0.87	0.89	0.77
Lysine (-)	145.0968	13.02	0.036	<0.001	0.91	0.97	0.88
Oxovaleric acid (-)	115.0384	9.90	<0.001	<0.001	0.81	0.91	0.74
Pentoses (-)	149.0441	9.96	0.127	<0.001	0.93	0.84	0.78
**Phenylalanine (+)**	166.0865	9.74	0.003	<0.001	0.88	0.95	0.83
Tyrosine (+)	182.0815	11.62	<0.001	<0.001	0.79	0.92	0.73
Uridine (-)	243.0617	7.20	0.004	0.006	0.82	0.99	0.81
**Valine° (-)**	116.0700	10.19	<0.001	<0.001	0.82	0.92	0.75
		increasing trend					
Glutamine° (+)	147.0767	13.63	<0.001	0.003	1.17	1	1.13
Glycine° (+)	76.0400	14.2	<0.001	<0.001	1.89	1	1.85
Hydroxydecanoic acid (-)	187.1329	9.45	<0.001	<0.001	1.59	1.68	2.68
**Indoxyl sulphate (-)**	212.0013	9.13	0.067	<0.001	1.35	1.76	2.38
PC C40:7 (+)	832.5865	5.14	0.641	<0.001	1.04	1.34	1.4
**Trimethylamine-*N*-oxid (+)**	76.0764	11.88	0.022	<0.001	1.99	1.3	2.59

*detailed information about category of identification according to Sumner et al. (39) is provided in S3 Table

** unadjusted p-values from paired t-test,

^°^ metabolites identified with explicitly search

***ratio based on mean-values.

Interpretation of the identified metabolites was based on trend-patterns which were assigned to one of four different pattern groups. 9 out of the 36 metabolites showed unidirectional trends in intensities (either increasing or decreasing): trimethylamine-*N*-oxide (TMAO) and indoxyl-sulfate, glycine and PC C40:7 (phosphatidylcholine) (increased after surgery), or branched chain amino acids (BCAA) choline, tyrosine, alanine and phenylalanine (decreased after surgery) (Table 2).

Examples for “V-pattern” are the following metabolites: creatine, ornithine, tryptophan and LysoPC C16:1, LysoPC C18:2. Examples for “Ʌ-pattern” are hydroxyisobutyric acid and acetylglycine (Table 3). All significantly changed metabolites are summarized in Tables 2 and 3.

**Table 3:** Bidirectional trends of changes in the intensities (peak-AUC) of identified metabolites before and after bariatric surgery, metabolites in bold have previously been associated with CVR.

Metabolite* (Ionization-mode)	MzMed	RtMed	p-value** PRE-POST	p-value** PRE-FU	Ratio *** PRE,POST	Ratio POST, FU	Ratio PRE,FU
		V-pattern					
Creatine (+)	132.0771	12.19	<0.001	<0.001	0.66	1.09	0.72
LysoPC C16:1 (+)	494.3249	4.83	0.077	0.288	0.85	1.29	1.09
LysoPC C18:2 (+)	520.3407	5.13	<0.001	0.863	0.68	1.48	1.01
**Ornithine (-)**	131.0815	12.54	0.004	0.019	0.83	1.34	1.11
PC C34:3 (+)	756.5550	5.27	<0.001	0.370	0.66	1.44	0.95
PC C36:5 (+)	780.5550	5.01	<0.001	0.006	0.67	1.21	0.81
PC C36:6 (+)	778.5389	4.61	<0.001	0.809	0.48	2.06	0.98
Sarcosine (-)	88.0386	11.27	<0.001	<0.001	0.78	1.1	0.86
Tryptophan (+)	205.0973	9.83	<0.001	<0.001	0.74	1.1	0.81
Uracil (+)	113.0351	6.98	<0.001	<0.001	0.75	1.04	0.78
		Ʌ-pattern					
Acetylglycine (-)	116.0337	12.97	<0.001	<0.001	2.78	0.74	2.05
Arginine (+)	175.1193	12.15	0.620	0.233	0.97	1.08	1.05
Carnitine (+)	162.1127	10.88	0.004	0.515	1.19	0.86	1.03
Hydroxyisobutyric acid (-)	103.0387	12.10	<0.001	<0.001	3.3	0.21	0.71
Leu Pro (+)	229.1548	9.19	<0.001	0.180	1.64	0.55	0.9
LysoPE C20:4 (+)	502.2936	8.27	0.022	0.227	1.16	0.94	1.09
Pantothenic acid (-)	218.1025	12.69	0.001	0.270	1.52	0.75	1.14
PC C38:6 (+)	806.5705	5.21	<0.001	0.020	1.31	0.88	1.15
Pyroglutamic acid (-)	128.0337	12.89	0.002	0.038	1.18	0.93	1.1
Threonine (+)	120.0660	13.11	0.602	<0.001	1.04	0.75	0.79

*detailed information about category of identification according to Sumner et al. (39) is provided in S3 Table

** unadjusted p-values from paired t-test,

***ratio based on mean-values.

### Metabolites linked to clinical outcomes

The median weight reduction at follow-up was 37.7 kg (iQR: 16.25 kg). For relating weight-loss with metabolomics data, patients were allocated to a high weight loss (HWL) and low weight loss (LWL) group. The weight-loss ratio was calculated as: weight one year post surgery/weight at baseline = FU/PRE with a weight-loss median of 0.7. HWL was below the weight loss median and LWL was above the weight-loss median. From the originally identified metabolites, creatinine, ornithine, arginine and valine were significantly lower in the HWL group compared to the LWL group (see S2 and S3 Figs).

Out of the 24 patients having type 2 diabetes at baseline, 9 patients were having a diabetes remission after one year. Diabetes remission was defined as an HbA1c below 48 mmol/mol (6.5%) without pharmacological treatment. Patients with a diabetes remission were younger (42 ± 8 years vs. 55±10 years), had shorter diabetes duration (6± 7 years vs. 11± 7 years) but higher weights pre-surgery (138 ±19 kg vs 124 ± 22 kg) compared to non-remission patients (S1 Fig). However, patients with diabetes remission also had a significantly larger weight reduction in the first year after bariatric surgery.

**Fig 5:**
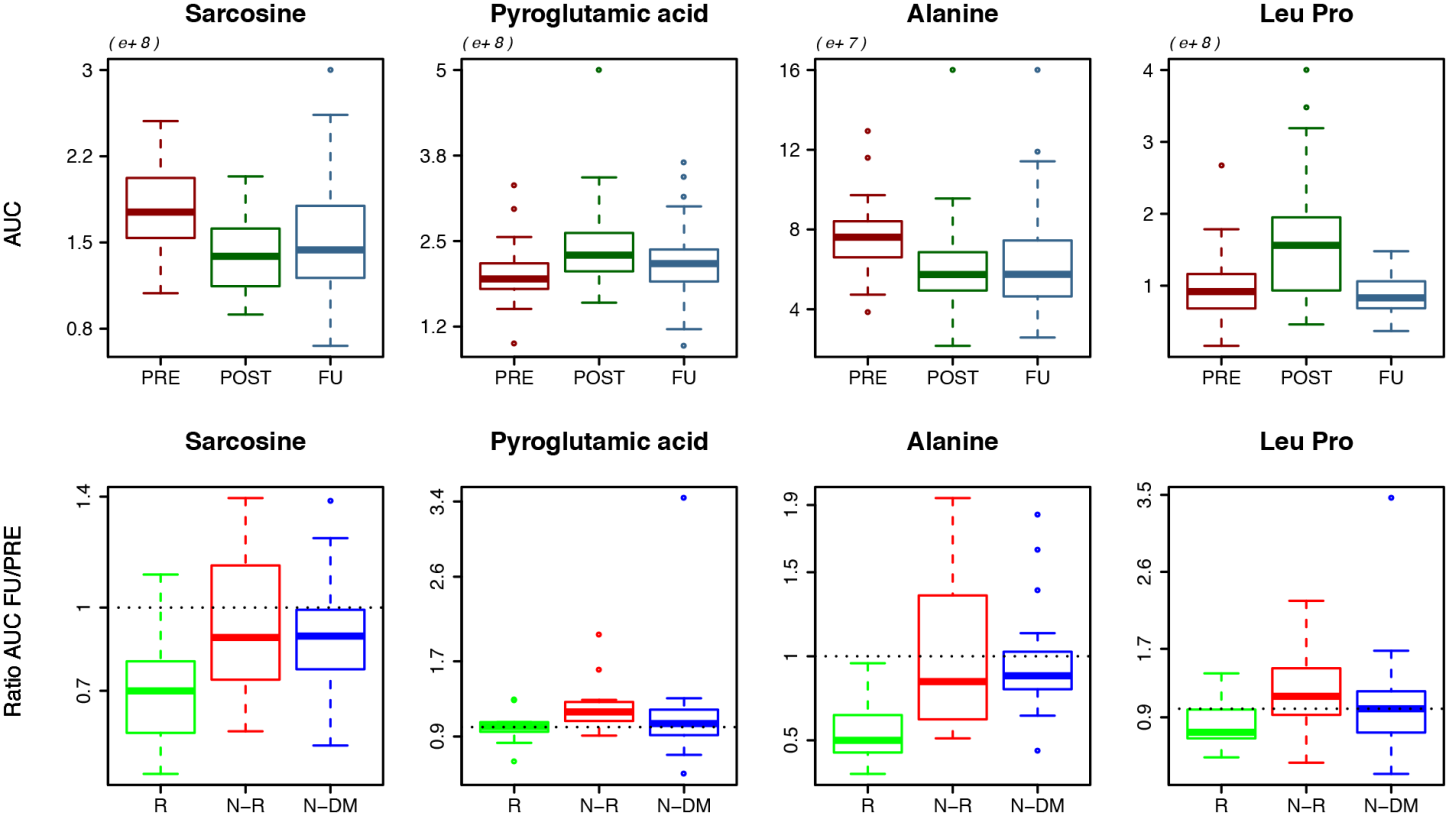
Metabolites showing a significant decline (FU/PRE) in diabetes remission (R) patients compared to non-remission (N-R). Above: Metabolite changes over time, below: different metabolite levels for diabetes remission (R), non-remission (N-R) and non-diabetes patients (N-DM)

Four originally identified metabolites showed a significantly larger decline in patients with diabetes remission compared to patients without diabetes remission. (Fig 5) (Sarcosine p = 0.031, pyroglutamic acid: p = 0.044, alanine: p = 0.005 and leucyl-proline: p = 0.049, summary of values see S5 Table).

## Discussion

In this study, we used an untargeted metabolomics approach to identify a set of metabolites which characterizes short- as well as long-term changes after bariatric surgery. In addition, we also investigated known metabolites which are relevant in the pathogenesis of cardiovascular diseases or which have previously been shown to be associated with cardiovascular outcome. In total, we were able to identify 36 metabolites. Their changes over time are best described as trend patterns. Metabolites which display unidirectional trends of increasing or decreasing intensities are more likely to be of interest for future studies.

The considerable difference in patterns between short and long-term changes highlights the importance of repeated measurements of metabolic patterns after an intervention. Focusing metabolic analysis on a single point in time can easily lead to false conclusions on the relevance of metabolites as potential biomarkers. Our analyses underline the need for a comprehensive analysis of short-term as well as long-term changes in order to gain a more complete picture of the metabolic changes induced by bariatric surgery. We put a special focus on metabolites that are known to be associated with cardiovascular disease and CVR factors (including diabetes) such as amino-acids (including BCAA), phospholipids (PCs), phenylalanine, TMAO and indoxyl sulfate.

BCAA intensities showed a decreasing trend after bariatric surgery similar to most other amino acids. Previous studies have described lower levels of BCAA to be associated with improved glucose metabolism and insulin sensitivity [20,21,29,34,58–62]. Data from the Framingham (Heart) Offspring Study demonstrated an association of high levels of BCAA with an increased risk for cardiovascular disease [56,57]. Our results also showed an increasing trend in glycine levels which has previously been described as a short-term effect of bariatric surgery [35] and inversely related to type 2 diabetes [63,64]. But we did not observe any difference between diabetes-remission and non-remission patients. In general our results for BCAA and other amino acids support the importance of these metabolites in the improved metabolism after bariatric surgery.

In our analysis all identified PCs were strongly influenced by bariatric surgery but pattern differed considerably over time. For example, PC38:6 has been associated with an increased risk of diabetes [65]. Our data show a short-term increase of PC38:6 but the increase was lower at the long-term visit relative to baseline, resulting in a Ʌ-pattern. An explanation for changes in the PC species that rise shortly after surgery is that individuals are in a significant catabolic state and lipids are mobilized from fat. Another possible cause for the short-term increase might be the dietary pattern shortly after bariatric surgery or the surgical procedure itself causing catabolic states [66,67]. Also, our short-term results are in line with a recently published study which showed an increase of PC38:6 in the first 42 days after bariatric surgery [68]. These Ʌ-patterns clearly demonstrate the relevance of long-term metabolic monitoring after bariatric surgery.

For other PCs such as PC36:5 we found a V-pattern or a steady increase (PC40:7), both metabolites have been shown to be inversely associated with coronary artery disease and mortality [69].

Phenylalanine, choline and tyrosine levels decreased after surgery, an effect that was sustained after one year at follow-up, similar to previous findings that have described increased phenylalanine and tyrosine as biomarkers for CVR [57,70].

TMAO and indoxyl-sulfate were also among the identified metabolites and are known cardiovascular markers [71]. TMAO is derived from the gut bacterial metabolism of choline, it has been shown to be directly associated with cardiovascular outcome and was suggested as a potential metabolic link between gut microbiome and cardiovascular diseases [56,71,72]. In contrast to previous studies, our results showed a significant increase of TMAO and indoxyl-sulphate similar to results of a bariatric study in rats [71]. One possible explanation for the increase of TMAO in patients could be a surgery-induced change in gut microbiome composition. Alternatively, carnitine can induce formation of TMAO [73] and carnitine is often promoted as a weight loss inducing supplement. Although the supplements recommended in this study did not contain carnitine we cannot exclude the possibility that patients were taking carnitine on their own. This interpretation is limited since we neither assessed dietary details to adjust the TMAO analysis to dietary composition nor did we collect stool samples to analyze gut microbiome composition. However, all patients underwent standardized nutritional counseling and received the same supplementation recommendations (S4 Table) following international guidelines [66,67]. Whether or not TMAO is a useful marker for CVR factors in patients undergoing bariatric surgery needs to be studied further.

Our in-depth analyses of patients with established diabetes mellitus confirm previously described predictors of diabetes remission after bariatric surgery such as age and diabetes duration at the time of the surgery [74]. Furthermore, our metabolomics analysis demonstrates that patients having a diabetes remission after one year showed larger declines in amino acids levels of alanine, proline and leucine as well as in the glycine metabolite sarcosine and the glutaminic acid derivate pyroglutamic acid. Previous studies have demonstrated an association of increased levels of BCAA and aromatic amino acids with diabetes incidence [75]. Our study adds information to the few available human data on diabetes and associated levels of sarcosine, leucyl-proline and pyroglutamic acids.

The success of an untargeted metabolomics approach is largely determined by the applied data processing workflow. Our four step untargeted metabolomics approach included data processing and statistical selection of metabolic feature and was successfully used in this clinical study. Data processing included filtering and time dependent drift correction on QC-intensities, measured by LC-HRMS. The use of the same pooled QC sample to observe the LC-HRMS measurement stability over time is a commonly used technique [76,77]. For QC based drift correction we used quantile regression which is often used for data modelling with heterogeneous conditional distributions [51]. Quantile regression has already been successfully used in metabolomics for drift correction and baseline alignment [78–81]. A smoothing step by a locally adaptive regression technique was applied because of the high variability of metabolic features. By using quantile regression techniques, the distributions of data quantiles were modelled separately, when dependencies were not equal in different quantiles. We used a nonparametric quantile regression to suit the conditional quantile functions, which fits a piecewise cubic polynomial with the number of one third of available data-points knots (breakpoints) arranged at the quantiles of the QCs-signals [51]. An amelioration of QC-variance was achieved by drift correction: the median CV of QC-intensities from all 924 metabolic features was reduced from 0.2 to 0.1.

The selection of metabolic features was based on significant p-values of univariate paired t-tests and metabolic feature importance of Random Forest (RF), a well-established methodology, in the processing of metabolomics data [77,82–86]. RFs are also better suited for metabolomics because of reduced overfitting and improved model prediction [82,83,87,88] compared to other supervised classification methods such as PLS-DA. In our study, RFs clearly indicated clusters of samples taken before and after bariatric surgery (MDS-Plot unsupervised RF). Remaining overlaps of clusters could be caused by individual responses to bariatric surgery.

While RFs and quantile regression are commonly used techniques in the field of metabolomics, we successfully applied a novel data-driven approach by combining quantile regression and RFs to clinical data.

## Conclusion

In summary, our data revealed both short-term and long-term metabolic effects of bariatric surgery in humans. The different identified patterns highlight the importance of repeated measurements over longer time periods in order to obtain a comprehensive understanding of the metabolic effects of bariatric surgery. Our study provides a better insight to changes in previously discussed metabolic CVR factors and to potential metabolic markers for diabetes remission. Our results also indicate that some metabolites might behave differently in patients with bariatric surgery relative to other risk cohorts. For future more personalized medicine the metabolic effects of clinical interventions need to be understood in more detail.

## Supporting Information

S1 Fig Non-remission patients (n) are significantly older than remission (c) (42(9) years vs *(docx)* 55(9)). Nd = non-diabetes.
(DOCX)

S2 Fig Median weight reduction. (DOCX)

S3 Fig Metabolites with significant changes between high and low-weight loss patients. (DOCX)

S1 Table Demographical and clinical raw data from the study population. (XLSX)

S2 Table Final, processed data used for statistical analysis and annotated and identified metabolites. (XLSX)

S3 Table Detailed information about category of identification. (XLSX)

S4 Table Nutritional information: Supplements after bariatric surgery. (DOCX)

S5 Table Metabolites showing significant changes between diabetes remission and non-remission. (DOCX)

S1 Text Word-file, containing additional tables and figures of metabolites linked to clinical outcomes. (DOCX)

S2 Text Word-file, containing research plan of clinical study. (DOCX)

S3 Text Power-point file containing the consort-diagram of the clinical study. (PPTX)
