# Supplementary material for: An Untargeted Metabolomics Approach to Characterize Short-Term and Long-Term Metabolic Changes after Bariatric Surgery
Source: PLoS One. 2016 Sep 1;11(9):e0161425. doi: 10.1371/journal.pone.0161425 (PMC5008721; doi:10.1371/journal.pone.0161425)
Supplement: S1 Fig — Nd = non-diabetes. (DOCX) [file pone.0161425.s001.docx]

**S1 Fig**

S1 Fig: Non-remission patients (n) are significantly older than remission (c) (42(9) years vs 55(9)). Nd=non-diabetes
