# Supplementary material for: An Untargeted Metabolomics Approach to Characterize Short-Term and Long-Term Metabolic Changes after Bariatric Surgery
Source: PLoS One. 2016 Sep 1;11(9):e0161425. doi: 10.1371/journal.pone.0161425 (PMC5008721; doi:10.1371/journal.pone.0161425)
Supplement: S2 Fig — (DOCX) [file pone.0161425.s002.docx]

**S2 Fig**

S2 Fig: The median weight reduction seen at follow up was 37.7 kg (iQR: 16.25 kg). We calculated a weight-loss ratio (weight 1 year post surgery / weight at baseline: FU/ POST) and allocated subjects into a high weight loss (HWL) and low weight loss (LWL) group, if they were below or above the median of the weight-loss median of 0.7.
