# Supplementary figures and images for: An Untargeted Metabolomics Approach to Characterize Short-Term and Long-Term Metabolic Changes after Bariatric Surgery
Source: PLoS One. 2016 Sep 1;11(9):e0161425. doi: 10.1371/journal.pone.0161425 (PMC5008721; doi:10.1371/journal.pone.0161425)

**S3 Fig**


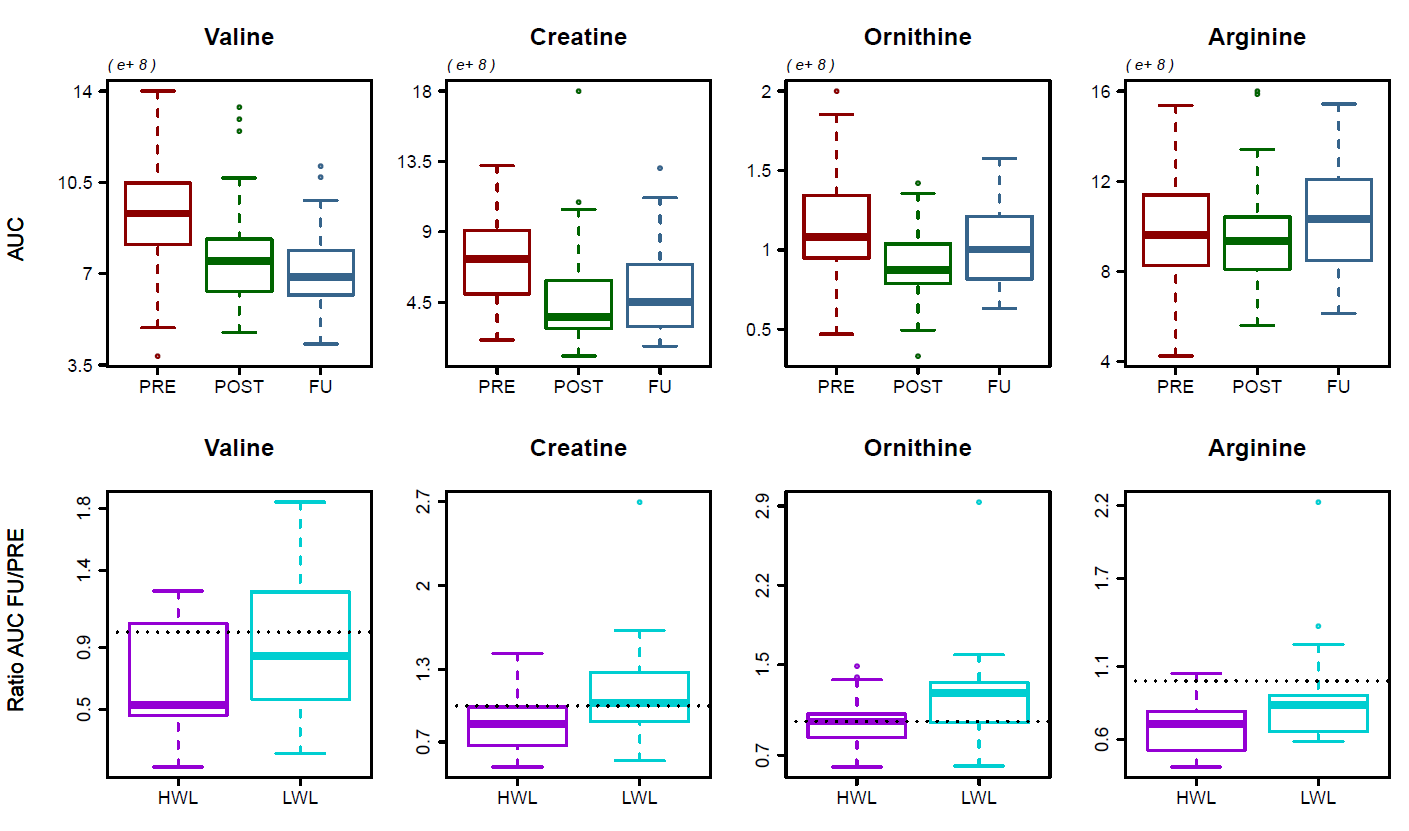


S3 Fig: Metabolites with significant changes between high and low-weight loss patients.

Supplement: S3 Fig — (DOCX) [file pone.0161425.s003.docx]
