# Supplementary material for: An Untargeted Metabolomics Approach to Characterize Short-Term and Long-Term Metabolic Changes after Bariatric Surgery
Source: PLoS One. 2016 Sep 1;11(9):e0161425. doi: 10.1371/journal.pone.0161425 (PMC5008721; doi:10.1371/journal.pone.0161425)
Supplement: S4 Table — (DOCX) [file pone.0161425.s007.docx]

**S4 Table**

S4 Table: Nutritional information: Supplements after bariatric surgery. All subjects underwent standardized nutritional counseling and received the same supplementation recommendations according to the guidelines (German S3 guideline on obesity and surgery)^[[1]](#footnote-1)^

| **Supplement** | **Amount** | **Frequency** |
| --- | --- | --- |
| Calcium-Carbonate (-Citrate) | 1.5 g | daily |
| Iron III iv. *or* | 200 mg | every 3-6 months |
| Iron II p.o. | 100-200 mg |  |
| Vitamine D3 p.o. *(OleovitD3)* *or* | 1200 IU |  |
| Vitamine D3 i.m. | 300 000 IU | every 3-6 months |
| Vitamine B12 i.m. *(Erycytol)* | 1000 μg | every 3-6 months |
| Vitamine B combination |  | twice a week |
| Multivitamin micronutrient supplement |  | daily |

1. [*http://www.adipositas-gesellschaft.de/fileadmin/PDF/Leitlinien/ADIP-6-2010.pdf*](http://www.adipositas-gesellschaft.de/fileadmin/PDF/Leitlinien/ADIP-6-2010.pdf) [↑](#footnote-ref-1)
