# Supplementary material for: An Untargeted Metabolomics Approach to Characterize Short-Term and Long-Term Metabolic Changes after Bariatric Surgery
Source: PLoS One. 2016 Sep 1;11(9):e0161425. doi: 10.1371/journal.pone.0161425 (PMC5008721; doi:10.1371/journal.pone.0161425)
Supplement: S5 Table — (DOCX) [file pone.0161425.s008.docx]

**S5 Table**

S5 Table: Metabolites showing significant changes (ratio FU/PRE) between diabetes remission and non-remission, p-values from paired t-test remission vs non-remission

|  | DBR | mean | sd | median | IQR | min | max | p-value |
| --- | --- | --- | --- | --- | --- | --- | --- | --- |
| Sarcosine | remission | 0.696 | 0.237 | 0.700 | 0.258 | 0.400 | 1.119 | 0.031 |
|  | non-remission | 0.943 | 0.276 | 0.892 | 0.412 | 0.554 | 1.395 |  |
|  | non-dm | 0.893 | 0.220 | 0.897 | 0.179 | 0.503 | 1.384 |  |
| Pyroglutamic acid | remission | 1.006 | 0.205 | 1.006 | 0.102 | 0.631 | 1.293 | 0.044 |
|  | non-remission | 1.217 | 0.272 | 1.161 | 0.202 | 0.910 | 1.985 |  |
|  | non-dm | 1.139 | 0.577 | 1.038 | 0.226 | 0.502 | 3.437 |  |
| Alanine | remission | 0.564 | 0.205 | 0.500 | 0.223 | 0.300 | 0.959 | 0.005 |
|  | non-remission | 0.984 | 0.439 | 0.849 | 0.737 | 0.512 | 1.941 |  |
|  | non-dm | 0.963 | 0.332 | 0.884 | 0.218 | 0.436 | 1.842 |  |
| Leu Pro | remission | 0.844 | 0.298 | 0.725 | 0.337 | 0.431 | 1.413 | 0.049 |
|  | non-remission | 1.187 | 0.509 | 1.145 | 0.545 | 0.369 | 2.262 |  |
|  | non-dm | 1.062 | 0.671 | 1.000 | 0.460 | 0.239 | 3.463 |  |
