# Supplementary material for: An Untargeted Metabolomics Approach to Characterize Short-Term and Long-Term Metabolic Changes after Bariatric Surgery
Source: PLoS One. 2016 Sep 1;11(9):e0161425. doi: 10.1371/journal.pone.0161425 (PMC5008721; doi:10.1371/journal.pone.0161425)
Supplement: S1 Text — (DOCX) [file pone.0161425.s009.docx]

**S1 Text**

Table A: 19 patients enrolled in Swiss study-center (St. Gallen) and 25 patients in Austrian centre (Graz), 24 patients had type 2 diabetes at baseline, whereas 9 of them could benefit from a complete diabetes remission after one year.

| DMR | Graz | St.Gallen | Sum |
| --- | --- | --- | --- |
| Non-DM | 14 | 6 | 20 |
| Diabetes remission | 4 | 5 | 9 |
| Non-or partial remission | 7 | 8 | 15 |

Table B: Metabolites showing significant changes (ratio FU/PRE) between high-weight loss patients (HWL) and low weight loss patients (LWL)

|  |  | mean | sd | median | IQR | min | max | p-value |
| --- | --- | --- | --- | --- | --- | --- | --- | --- |
| Valine | HWL | 0.662 | 0.338 | 0.528 | 0.520 | 0.127 | 1.266 | 0.043 |
|  | LWL | 0.924 | 0.482 | 0.845 | 0.636 | 0.212 | 1.842 |  |
| Creatine | HWL | 0.852 | 0.241 | 0.847 | 0.307 | 0.492 | 1.436 | 0.011 |
|  | LWL | 1.143 | 0.444 | 1.024 | 0.405 | 0.543 | 2.693 |  |
| Ornithine | HWL | 1.002 | 0.238 | 0.999 | 0.196 | 0.598 | 1.485 | 0.048 |
|  | LWL | 1.227 | 0.456 | 1.253 | 0.350 | 0.606 | 2.935 |  |
| Arginine | HWL | 0.697 | 0.178 | 0.707 | 0.233 | 0.411 | 1.051 | 0.029 |
|  | LWL | 0.893 | 0.359 | 0.834 | 0.241 | 0.585 | 2.225 |  |
|  |  |  |  |  |  |  |  |  |

Table E: Unidirectional trends of changes in the intensities (peak-AUC) of identified metabolites before and after bariatric surgery, metabolites in bold have previously have been associated with CVR.

| **Metabolite** | **MzMed** | **RtMed** | **p-value** PRE-POST** | **p-value** POST-FU** | **p-value** PRE-FU** | **Ratio PRE,POST** | **Ratio POST, FU** | **Ratio PRE,FU** |
| --- | --- | --- | --- | --- | --- | --- | --- | --- |
| **(Ionization-mode)** |  |  |  |  |  |  |  |  |
|  |  | decreasing trend |  |  |  |  |  | |
| **Alanine (+)** | 90.0556 | 12.2 | <0.001 | 0.468 | 0.019 | 0.8 | 1 | 0.85 |
| **Choline (+)** | 104.1076 | 10.06 | <0.001 | 0.397 | 0.003 | 0.74 | 1 | 0.79 |
| **Leucine/isoleucine° (+)** | 132.1022 | 9.45 | 0.003 | 0.036 | <0.001 | 0.87 | 0.89 | 0.77 |
| Lysine (-) | 145.0968 | 13.02 | 0.036 | 0.424 | <0.001 | 0.91 | 0.97 | 0.88 |
| Oxovaleric acid (-) | 115.0384 | 9.9 | <0.001 | 0.089 | <0.001 | 0.81 | 0.91 | 0.74 |
| Pentoses (-) | 149.0441 | 9.96 | 0.127 | <0.001 | <0.001 | 0.93 | 0.84 | 0.78 |
| **Phenylalanine (+)** | 166.0865 | 9.74 | 0.003 | 0.247 | <0.001 | 0.88 | 0.95 | 0.83 |
| Tyrosine (-) | 180.0658 | 11.28 | <0.001 | 0.95 | <0.001 | 0.79 | 0.92 | 0.73 |
| Uridine (-) | 243.0617 | 7.2 | 0.004 | 0.876 | 0.006 | 0.82 | 0.99 | 0.81 |
| **Valine° (-)** | 116.07 | 10.19 | <0.001 | 0.118 | <0.001 | 0.82 | 0.92 | 0.75 |
|  |  | increasing trend |  |  |  |  |  | |
| Glutamine° (-) | 145.0603 | 11.95 | <0.001 | 0.459 | 0.003 | 1.17 | 1 | 1.2 |
| Glycine° (-) | 74.0231 | 11.69 | <0.001 | 0.162 | <0.001 | 1.24 | 1 | 1.35 |
| Hydroxydecanoic acid (-) | 187.1329 | 9.45 | <0.001 | 0.002 | <0.001 | 1.59 | 1.68 | 2.68 |
| **Indoxyl sulphate (-)** | 212.0013 | 9.13 | 0.067 | 0.001 | <0.001 | 1.35 | 1.76 | 2.38 |
| PC C40:7 (+) | 832.5865 | 5.14 | 0.641 | 0.002 | <0.001 | 1.04 | 1.34 | 1.4 |
| **Trimethylamine-*N*-oxid (+)** | 76.0764 | 11.88 | 0.022 | 0.169 | <0.001 | 1.99 | 1.3 | 2.59 |

*detailed information about category of identification according to Sumner et al. (39) is provided in S3 Table

** unadjusted p-values from paired t-test, ° metabolites identified with explicitly search ***ratio based on mean-values

Table F: Bidirectional trends of changes in the intensities (peak-AUC) of identified metabolites before and after bariatric surgery, metabolites in bold have previously have been associated with CVR.

| **Metabolite** | **MzMed** | **RtMed** | **p-value** PRE-POST** | **p-value** POST-FU** | **p-value** PRE-FU** | **Ratio PRE,POST** | **Ratio POST, FU** | **Ratio PRE,FU** |
| --- | --- | --- | --- | --- | --- | --- | --- | --- |
| **(Ionization-mode)** |  |  |  |  |  |  |  |  |
|  |  | V-pattern |  |  |  |  |  | |
| Creatine (+) | 132.0771 | 12.19 | <0.001 | 0.498 | <0.001 | 0.66 | 1.09 | 0.72 |
| LysoPC C16:1 (+) | 494.3249 | 4.83 | 0.077 | 0.018 | 0.288 | 0.85 | 1.29 | 1.09 |
| LysoPC C18:2 (+) | 520.3407 | 5.13 | <0.001 | <0.001 | 0.863 | 0.68 | 1.48 | 1.01 |
| **Ornithine (-)** | 131.0815 | 12.54 | 0.004 | 0.019 | 0.019 | 0.83 | 1.34 | 1.11 |
| PC C34:3 (+) | 756.555 | 5.27 | <0.001 | <0.001 | 0.37 | 0.66 | 1.44 | 0.95 |
| PC C36:5 (+) | 780.555 | 5.01 | <0.001 | 0.048 | 0.006 | 0.67 | 1.21 | 0.81 |
| PC C36:6 (+) | 778.5389 | 4.61 | <0.001 | <0.001 | 0.809 | 0.48 | 2.06 | 0.98 |
| Sarcosine (-) | 88.0386 | 11.27 | <0.001 | 0.124 | <0.001 | 0.78 | 1.1 | 0.86 |
| Tryptophan (+) | 205.0973 | 9.83 | <0.001 | 0.093 | <0.001 | 0.74 | 1.1 | 0.81 |
| Uracil (+) | 113.0351 | 6.98 | <0.001 | 0.574 | <0.001 | 0.75 | 1.04 | 0.78 |
|  |  | Ʌ-pattern |  |  |  |  | | |
| Acetylglycine (-) | 116.0337 | 12.97 | <0.001 | 0.025 | <0.001 | 2.78 | 0.74 | 2.05 |
| Arginine (+) | 175.1193 | 12.15 | 0.62 | 0.102 | 0.233 | 0.97 | 1.08 | 1.05 |
| Carnitine (+) | 162.1127 | 10.88 | 0.004 | 0.022 | 0.515 | 1.19 | 0.86 | 1.03 |
| Hydroxyisobutyric acid (-)# | 103.0387 | 12.1 | <0.001 | <0.001 | <0.001 | 3.3 | 0.21 | 0.71 |
| Leu Pro (+) # | 229.1548 | 9.19 | <0.001 | <0.001 | 0.18 | 1.64 | 0.55 | 0.9 |
| LysoPE C20:4 (+) | 502.2936 | 8.27 | 0.022 | 0.393 | 0.227 | 1.16 | 0.94 | 1.09 |
| Pantothenic acid (-) | 218.1025 | 12.69 | 0.001 | 0.027 | 0.27 | 1.52 | 0.75 | 1.14 |
| PC C38:6 (+) | 806.5705 | 5.21 | <0.001 | 0.038 | 0.02 | 1.31 | 0.88 | 1.15 |
| Pyroglutamic acid (-) | 128.0337 | 12.89 | 0.002 | 0.156 | 0.038 | 1.18 | 0.93 | 1.1 |
| Threonine (+) | 120.066 | 13.11 | 0.602 | 0.006 | <0.001 | 1.04 | 0.75 | 0.79 |

Figure E: Multidimensional Scaling Plot of unsupervised Random Forests with initial 923 metabolic features

Figure F: Multidimensional Scaling Plot of unsupervised Random Forests with 36 identified Metabolites


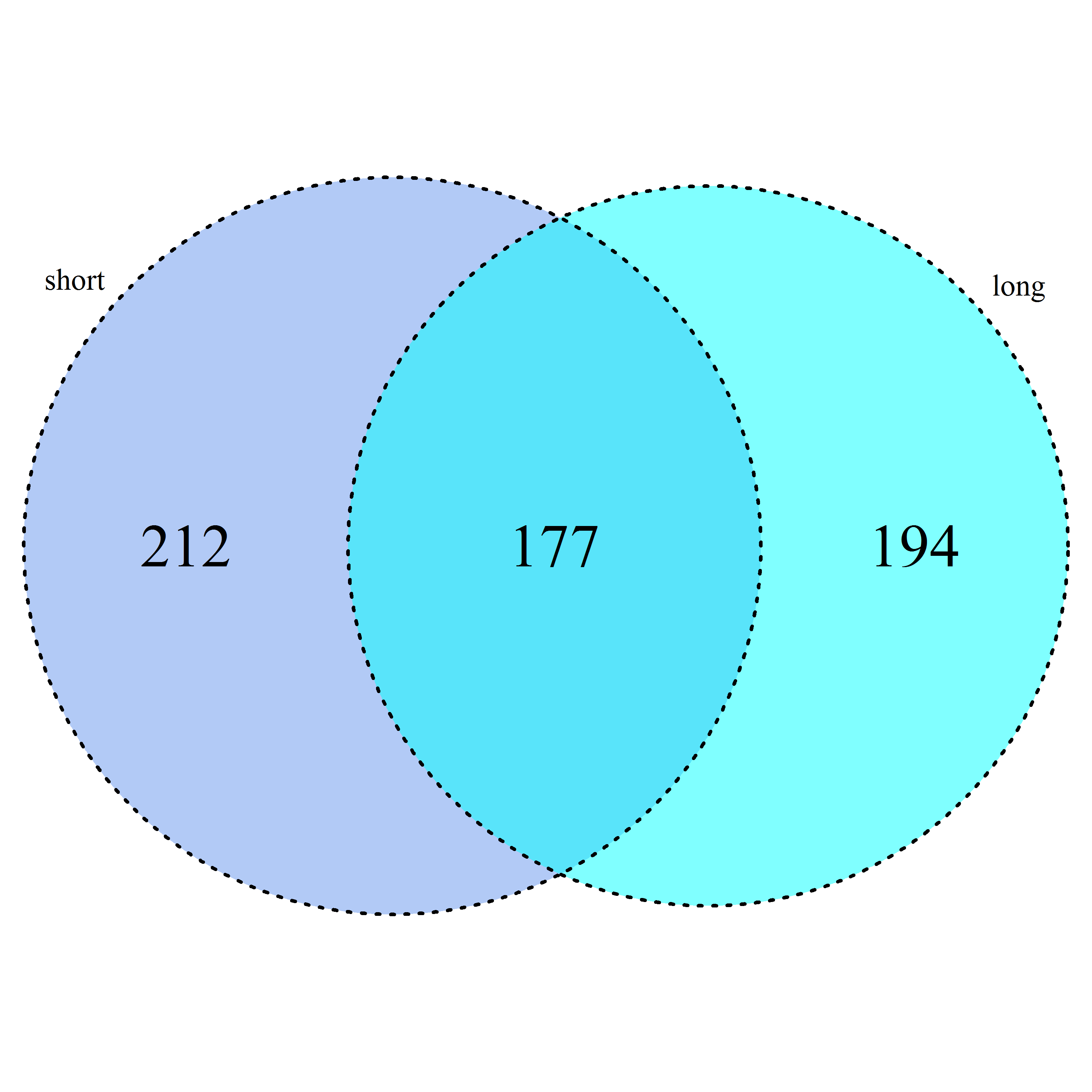


Figure G: Number of metabolic features with significant differences between PRE and POST (short-term) and PRE and FU (long-term). The intersection was presented as the main result.


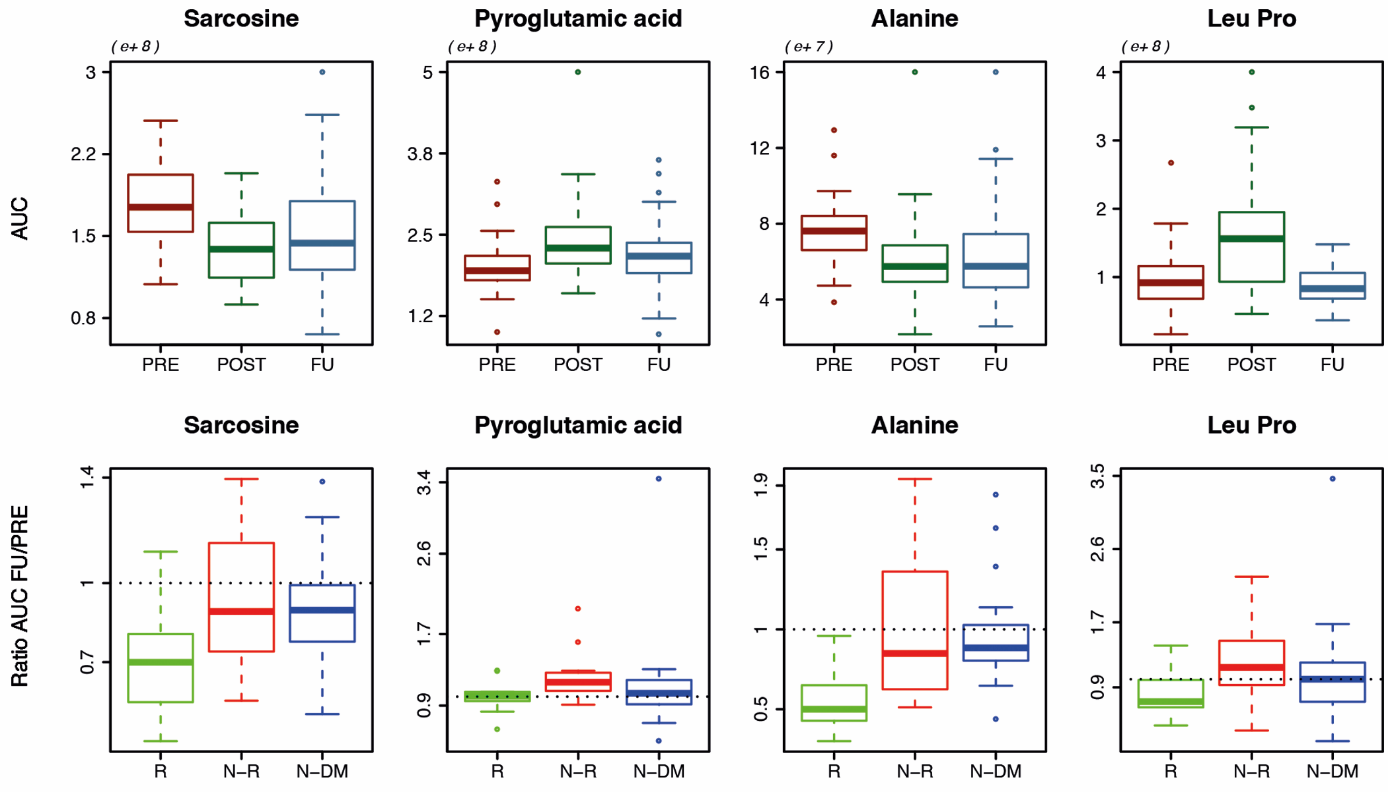


Figure H: Metabolites showing significant changes (ratio FU/PRE) between patients with diabetes-remission (R) and non-remission (N-R), non-diabetes are shown additionally (N-DM). Significances are listed in table B above
