# Supplementary material for: An Untargeted Metabolomics Approach to Characterize Short-Term and Long-Term Metabolic Changes after Bariatric Surgery
Source: PLoS One. 2016 Sep 1;11(9):e0161425. doi: 10.1371/journal.pone.0161425 (PMC5008721; doi:10.1371/journal.pone.0161425)
Supplement: S2 Text — (DOCX) [file pone.0161425.s010.docx]

**S2_Text**

**EFSD/MSD European Studies on Islet Cell Function and Survival:**

**Clinical Research Programme**

**Mechanisms of restoration of beta cell function in relation to adipoinsular and enteroinsular axes after gastric bypass surgery in severely obese patients with type 2 diabetes**

**Double-centre, prospective, controlled, longitudinal open study**

**Research Plan**

# A. Introduction

## 1. Objective

Bariatric operations such as the gastric bypass procedure provide a unique *in vivo* model of improvement of pathological beta cell function. The presented double-centre study aims to comprehensively investigate different aspects of beta cell function in patients with type 2 diabetes (T2DM) with a wide range of disease duration after gastric bypass. In parallel, our project will address the aspects of changes in enteroinsular and adipoinsular axes as well as the early and late changes of other defined parameters after gastric bypass surgery.

## 2. Background and current status of research in the proposed field of the study

Epidemiological evidence confirms that weight loss significantly decreases the risk for development of type 2 diabetes [1]. Morbid obesity per se is associated with profound insulin resistance and subsequent hypersecretion of insulin but the dynamics of beta cell function are still preserved [2]. On the other hand, overt diabetes is characterized by a progressive loss of beta cell glucose sensitivity [3]. In contrast to traditional weight reduction treatments, bariatric surgery has been proven not only to induce a sustained long-term weight loss but also to resolve type 2 diabetes in the majority of patients and it therefore represents an interesting model of weight loss and related changes in glucose metabolism [4]. However, resolution rates of diabetes after bariatric surgery clearly depend on the specific type of operation rather than on the amount of weight loss per se [5]. Bilio-pancreatic diversion (BPD) has consistently been found to show the highest remission rate (i.e. about 99%) followed by Roux-en-Y gastric bypass (85%), whereas purely restrictive procedures such as gastroplasty and gastric banding induce lower rates of diabetes resolution (72% and 48%; respectively). Together these findings point to distinct influences of different surgical procedures on glucose metabolism with those excluding the duodenum from the passage and enhancing the delivery of nutrients to the hindgut being the most effective.

Remarkably, in many cases the beneficial effect of bariatric surgery on glucose metabolism occurs within days after surgery, i.e. even before a significant weight loss has been achieved. However, in other cases glucose metabolism only gradually improved in parallel with the progression of weight loss until a complete remission has been achieved [6-8]. Observational clinical studies suggest that the chance of recovery from T2DM due to surgery decreases with increasing duration of the disease [9]. Though, surgery-induced remission of T2DM is also not uncommon in patients with long standing diabetes. Thus, strong predictors for the course of T2DM after gastric bypass surgery are still lacking which may result from insufficient preoperative metabolic characterisation of T2DM patients in many foregoing observational studies.

Mechanisms which lead to a resolution of T2DM following BDP and gastric bypass surgery are incompletely understood yet. A substantial improvement of insulin sensitivity and a decrease in absolute insulin secretion in combination with improvement of beta cell glucose sensitivity have been shown in previous studies [10, 11] The improvement of insulin sensitivity has been attributed to an enhanced insulin signalling in muscle [12] and changed expression of genes regulating glucose and lipid metabolism [13] within skeletal muscle tissue along with a depletion of intramyocellular fat stores [14].

In regard to beta cell function it has been shown that glucose sensitivity assessed by an oral glucose tolerance test (OGTT) markedly improved within one week after a BPD operation [10, 15] and that this improvement continued over a period of at least two years [16]. The improvement of beta cell function has been attributed to enhanced secretion of incretins, especially glucagon-like peptide-1 (GLP-1), and also to reduced release of yet to be identified anti-incretins resulting from the exclusion of the nutrient flow through the proximal intestine [17, 18]. However, several studies [19-21] have also demonstrated a restoration of the acute insulin response (AIR) during an intravenous glucose tolerance test (IVGTT) in T2DM patients early after a BPD operation. This finding suggests that enhanced incretin secretion does not represent the only mechanism underlying the improvement of beta cell function because the release of incretins critically relies on the passage of glucose through the gastrointestinal tract that is not given during an IVGTT. In addition, a reduction of lipotoxicity has been proposed as another mechanism contributing to the improvement of beta cell function after surgery [20]. However it should also be noted that all of the above mentioned results on beta cell function are derived from studies examining the effects of BPD not gastric bypass procedures. Of note, BPD operations in contrast to the standard proximal gastric bypass procedure lead to a strong lipid malabsorption which may result in an especially pronounced reduction of beta cell lipotoxicity. Overall, it is not even clear yet whether the AIR to an intravenous glucose challenge is recovered in T2DM patients after gastric bypass surgery and thus, whether the improvement of beta cell function relies on mechanisms not depending on an enhanced release of incretin hormones [22, 23]. This question appears to be of particular importance when considering that gastric bypass represents a far more popular bariatric procedure than the BPD [24].

Besides above discussed mechanisms, changes of adipokines and inflammatory mediators from adipose tissue have been postulated to be responsible for long-term improvement in glucose metabolism after gastric bypass in relation to reduced fat mass [25]. The role of adipose-tissue derived factors in insulin resistance is widely acknowledged [26], however their relation to beta cell function is not clarified yet. Based on cell culture experiments and rodent models, experimental evidence exists that adipokines and inflammatory mediators directly interact with beta cell function through regulation of lipid and glucose metabolism in the beta cell (leptin, free fatty acids), through changes in insulin secretion (leptin, TNFα, adiponectin, free fatty acids, IL6), changes in insulin gene expression (leptin), or through modification of beta cell glucose and insulin sensitivity or beta cell apoptosis (leptin, adiponectin, IL6, TNFα) [27, 28]. An inflammatory process in islets from patients with T2DM as a response to metabolic stress with activation of the IL-1β pathway has been recently postulated to contribute to beta cell failure and enhanced apoptosis [29].

Clinical data supporting causal relationships within the adipoinsular axis are still lacking. Changes in beta cell function after bariatric surgery therefore represent a useful clinical model for investigation of adipoinsular axis. However, clinical studies dealing with changes of adipokines after bariatric surgery did not address beta cell function in parallel, and vice versa.

Plasma concentrations of leptin are consistently reported to decrease after bariatric surgery, already within 4 weeks [10, 30-32], its decline reflects long-term BMI reduction [31], but the correlation of leptin with beta cell function improvement was rarely investigated and the results are conflicting – there are both pros [10, 33] and contras [20]. An increase in plasma adiponectin after bariatric surgery is consistently found in the literature [32, 34-36], but again the relation to beta cell function has been rarely investigated with contradictory results [20, 33]. Similarly, other adipose-tissue derived cytokines and inflammatory factors (TNFα, IL6, MCP-1, hs-CRP, IL-1ra [receptor antagonist for IL1β], IL10) implicated in chronic low-grade inflammation associated with obesity, have been previously assessed after bariatric surgery [30, 37-39], but were never related to beta cell function.

Additionally, most of the above mentioned studies used to determine only circulating concentrations of adipokines and inflammatory mediators. Changes in adipose-tissue derived factors at the tissue level (mRNA expressions) in comparison with controls were not systematically investigated yet.

Moreover, differential responses of adipoinsular axis to bariatric surgery in relation to diabetic status have not been elucidated so far, the indices in literature are based on small numbers of patients included in the study [40] and need further evaluation.

Besides the serious limitations on the mechanistic knowledge of beta cell function recovery after bariatric surgery outlined above, it must be emphasized that the available experimental data on this issue almost exclusively derive from patients with a rather short duration of T2DM (< 6 years). On this background, the question arises whether surgery induced beta cell function improvement also occurs after longer duration of the disease which is expected to be associated with aggravated beta cell damage. Such an improvement would dramatically challenge the traditional view of an irreversible damage to beta cells in long standing T2DM.

# Specific Aims

## Zero hypothesis: There is no difference in terms of beta cell function in patients with short- and long-term history of T2DM following gastric bypass.

## Alternative hypothesis: There is a significant difference in terms of beta cell function in patients with short- and long-term history of T2DM following gastric bypass.

**Primary objective**:

Comparison of the changes in beta cell function following gastric bypass in patients with short- and long-term history of T2DM

**Primary outcome variable**:

Acute insulin response (AIR) to glucose during IVGTT will be used as a primary variable characterizing the beta cell function.

**Secondary objectives:**

- - - Assessment of the beta cell insulin response to oral and intravenous glucose loads early and 1 year after gastric bypass surgery including the quantification of the incretin effect using oral and isoglycaemic intravenous glucose infusion tests
    - Relation of early and long-term changes in beta cell function to changes in parameters of adipoinsular and enteroinsular axes in severely obese patients with and without T2DM
    - Comparison of the changes in adipoinsular and enteroinsular axes after weight loss achieved after gastric bypass with BMI-matched non-bariatric controls

**Secondary outcome variables**:

- OGTT calculations and modelling describing beta cell response to oral glucose load (see paragraph D.5. Calculations, page 7); Insulin and C-peptide areas under the curve (AUCs) during OGTT and isoglycaemic iv. glucose infusion quantifying the incretin effect.
- Plasma concentrations and adipose tissue expression of selected adipokines and inflammatory cytokines characterizing the adipoinsular axis
- Plasma concentrations of incretins GLP-1 and GIP and their time profiles during OGTT and isoglycaemic iv. glucose infusion.

# Preliminary data

*Study group justification*

In St. Gallen, approximately 160 patients per year undergo gastric bypass surgery, of which about 30% display T2DM. Thus, it is expected to ensure the recruitment of 40 T2DM patients and 15 non-diabetic patients over 1 year. In Graz, approximately 60 patients per year are submitted for gastric bypass surgery, a third of them displaying T2DM. Expecting the recruitment of 15 T2DM patients and 15 non-diabetic (non-DM) patients per year in Graz, the total number of surgery patients included in the study is 85 patients (55 T2DM, 30 non-DM). A drop-out rate during follow-up is estimated for 10-12%.

*Publications regarding gastric bypass and glucose metabolism – St. Gallen*

The applicant from St. Gallen has a 3.5 years experience in the clinical management of morbid obese patients undergoing bariatric surgery. He has published on several nutritional aspects bariatric surgery [41-43]. Furthermore, he has extensive experience in performing experimental clinical studies on glucose metabolism with a special focus of hypoglycemia counterregulation [44-49].

*Established analysis of cytokines, hormones, gene expression – Graz*

Medical University of Graz disposes of established laboratorial facilities which have validated the methods applied in the presented study. The multiplex flow-cytometric bead based ELISA assay (LUMINEX) has been introduced and validated for multiple measurements of small sample volumes and published in previous studies [50-53]. The immunochemical analysis of endocrine and metabolic parameters (ELISA, RIA, LIA) is available both for routine and research purposes, as well as the gene expression analysis via real-time PCR documented by several publication [54-60]

# Methodology

## Population

Bariatric group: 85 severely obese patients (55 T2DM patients and 30 non-diabetic [non-DM] patients) fulfilling the indication criteria for laparoscopic gastric bypass surgery including the personal wish to undergo gastric bypass will be consecutively recruited at the Obesity Outpatient Clinic, Medical University Graz, Austria and at Interdisciplinary Obesity Center in St. Gallen, Switzerland. In the subgroup of T2DM surgery patients, patients with wide range of disease duration will be included. Long-standing T2DM will be classified as the disease duration longer than 10 years at the time of surgery. Within the T2DM subgroup, the expected proportion of long-standing T2DM will be 40-50%. For the detailed recruitment proportions at both sites see Table 1.

Control group: 30 patients (15 T2DM patients and 15 non-DM patients) undergoing elective laparoscopic abdominal surgery in the absence of neoplastic disease, acute or chronic inflammatory disease will be consecutively recruited at both sites. The control group will be matched with bariatric group for age, sex and expected one-year postoperative BMI (BMI 27-35 kg/m^2^).

| Site | **Bariatric group (n=85)** | | **Control group (n=30)**  **(15 T2DM / 15 non-DM)** |
| --- | --- | --- | --- |
|  | **T2DM** | **non-DM** |  |
| Graz, Austria | 15 | 15 | 15 |
| St. Gallen, Switzerland | 40 | 15 | 15 |
| Total | 55 | 30 | 30 |

*Table 1. Recruitment proportions at both sites.*

## Protocol

This double-center, prospective, controlled, longitudinal open clinical trial will be performed at Department of Endocrinology and Nuclear Medicine, Medical University Graz, Austria and at Interdisciplinary Obesity Center, Kantonsspital St. Gallen, Switzerland. Both centers will follow the same protocol. Both bariatric and control group will be examined at baseline before surgery. During surgical procedure samples of subcutaneous (SAT) and visceral (VAT) adipose tissue will be obtained. In bariatric patients, repeated follow-up visits will be arranged at 1-2 weeks and 12 months after the surgery and a needle biopsy of SAT will be performed at 12-months follow-up. Control group will be examined at baseline only.

At baseline visit detailed medical history and concomitant medication, particularly diabetes duration and history of diabetes therapy will be recorded. To verify the information on diabetes history the patient’s family physician will be contacted by phone calls.

At baseline and all follow-up visits, patients and controls will be studied on three occasions after an overnight fast and procedures will be grouped as follows:

1. Biochemistry, anthropometry, vital signs and oral glucose tolerance test (visits 1,4,7)
2. Isoglycaemic intravenous glucose infusion, vital signs (visits 2,5,8)
3. Botnia clamp, vital signs (visits 3, 6 and 9) and needle biopsy of SAT (visit 9)

For study visit and procedure schedule see Table 2.

| **Groups** | **Procedure** | **Baseline** | | | **1-2 weeks** | | | **12 months** | | |
| --- | --- | --- | --- | --- | --- | --- | --- | --- | --- | --- |
|  |  | **V1** | **V2** | **V3** | **V4** | **V5** | **V6** | **V7** | **V8** | **V9** |
| Bariatric group  (n=85)  55 T2DM/30 non-DM | Medical history | x |  |  | x |  |  | x |  |  |
|  | Vital signs | x | x | x | x | x | x | x | x | x |
|  | Biochemistry | x |  |  | x |  |  | x |  |  |
|  | Anthropometry | x |  |  | x |  |  | x |  |  |
|  | OGTT | x |  |  | x |  |  | x |  |  |
|  | Isoglyc. iv.infusion |  | x |  |  | x |  |  | x |  |
|  | Botnia clamp |  |  | x |  |  | x |  |  | x |
|  | SAT needle biopsy |  |  |  |  |  |  |  |  | x |
| Control group  (non-bariatric abdominal surgery)  (n=30)  15 T2DM/15 non-DM | Medical history | x |  |  |  |  |  |  |  |  |
|  | Vital signs | x | x |  |  |  |  |  |  |  |
|  | Biochemistry | x |  |  |  |  |  |  |  |  |
|  | Anthropometry | x |  |  |  |  |  |  |  |  |
|  | OGTT | x |  |  |  |  |  |  |  |  |
|  | Isoglyc. iv.infusion |  | x |  |  |  |  |  |  |  |
|  | Botnia clamp |  |  | x |  |  |  |  |  |  |

*Table 2. Study visit and procedure schedule.*

## 3. Procedures

- **Anthropometry:** weight, height, BMI, waist circumference, waist-to-hip ratio, body composition estimation (fat mass, fat-free mass) using a dual energy X-ray absorptiometry (Lunar iDXA, GE).
- **Vital signs:** blood pressure, heart rate, body temperature
- **Biochemistry:** fasting glucose, free fatty acids, lipids, electrolytes, HbA1c
- **Oral glucose tolerance test (OGTT)** with 75g of glucose (3 hours) with measurements of plasma glucose levels at 10-min intervals. Blood samples for analysis of insulin, proinsulin, C-peptide, glucagon, GLP-1 and GIP concentrations will be taken at 0,15,30,45,60,90,120,150 and 180min to assess changes in glucose tolerance, as well as to characterize responses of the enteroinsular axis to oral glucose.
- **Isoglycaemic iv. glucose infusion** (3 hours). To quantify the incretin effect, the plasma glucose profile from OGTT will be reproduced by a variable iv. glucose 20% infusion. Plasma glucose will be measured at 5-10 min intervals, blood sampling for insulin, C-peptide, glucagon, GLP-1 and GIP measurements will be performed at 0,15,30,45,60,90,120,150 and 180min.
- **Botnia clamp (combined IVGTT and hyperinsulinaemic-euglycaemic clamp)**, which is a validated design for assessment of beta cell function and insulin sensitivity [61]. After an overnight fast and after baseline samples and SAT needle biopsy (at visit 6) have been obtained, 0.3 g/kg bodyweight of 20% glucose solution will be given at time 0 min. Blood samples for the measurement of plasma glucose, insulin and C-peptide will be obtained at -10, 0, 2, 4, 6, 8, 10, 20, 30, 40, 50 and 60min. At 60 min after the glucose bolus, hyperinsulinaemic-euglycaemic clamp will be started to evaluate insulin sensitivity. A priming dose followed by an infusion (40mU/m^2^/min) of short-acting human insulin will be applied for 120 min. Blood glucose will be clamped at the concentration of 5.0 mmol/l by a variable infusion of 20% glucose. Blood samples for measurement of plasma glucose concentrations will be obtained at 5 min interval, for measurement of insulin concentrations in 30 min intervals throughout the clamp. The mean amount of glucose infused during the last 60 min of euglycaemic clamp will be used to calculate the rate of whole-body glucose uptake.

At baseline and 180 min of the Botnia clamp blood samples for determination of cytokines and adipokines (leptin, adiponectin, TNFα, hsCRP, MCP-1, IL10, IL6, IL1ra) will be collected.

- **Adipose tissue biopsies.** Two types of biopsies will be collected during the study:
  - Intraoperative during bariatric (Bariatric group) and elective surgery (Control group) – subcutaneous (SAT) and visceral (VAT) adipose tissue. Tissue samples will be acquired by surgeon at the beginning of the surgery.
  - Needle biopsy of SAT performed at 12 months of follow-up in Bariatric group. Under local anesthesia (1% trimecain in a field block pattern) an incision (3-4 mm) will be made in the skin at the lower abdomen and a subcutaneous fat specimen (300 mg) will be obtained by needle aspiration.

Based on experimental evidence of Biobank of Medical University Graz, study-specific standard operation procedures will be developed to ensure standardized and quality-assured biopsy processing, storage and shipping at both sites.

All samples will be washed with 0.9% saline solution, divided into 10 or 3 aliquot portions by intraoperative or needle biopsies, respectively and immediately frozen in liquid nitrogen. Samples will be stored at – 80°C until use for RNA extraction and gene expression analysis using real-time PCR method. Gene expression of adipokines (adiponectin and leptin), cytokines (such as TNFα, MCP-1, IL6, IL10, IL1ra), macrophage marker CD68 or genes involved in glucose and fatty acid metabolism will be analyzed (genes may be added according to actual scientific knowledge).

A subset of samples collected in Graz will be fixed with formalin and subsequently with paraffin in form of blocks. This processing enables further histological analysis of adipocyte size or macrophage infiltration in adipose tissue.

## 4. Analytical procedures

- **Glucose concentration** in arterialized blood samples will be measured in duplicate, using two Beckman Glucose Analyzers 2 (Beckman Instruments Inc., Fullerton, CA).
- Lipids, electrolytes, HbA1c, hs-CRP and free fatty acids will be analyzed using established laboratory techniques routinely available at both sites
- **Insulin and C-peptide** will be analyzed using routinely available partly automated ELISA methods in the Laboratory for Endocrinology and Metabolism and the EndoGenLab at Medical University Graz, Austria
- **GLP-1 and GIP analysis.** Blood samples will be collected into pre-chilled tubes containing EDTA plus aprotinin. After centrifugation plasma samples will be frozen at -80 °C until analysis. Total GLP-1 and GIP levels will be determined by RIA and ELISA methods, respectively.
- **Adipokines and cytokines**: Plasma and serum samples will be frozen for the prospective analysis of cytokines as follows:
  - Adiponectin, leptin will be measured using commercially available enzymatic immunoassays (ELISA; Biovendor Research Products, Heidelberg, Germany)
  - TNF-alpha, IL6, IL10, MCP-1, IL1ra will be analyzed using commercially available multiplexed ELISA assays (R&D Systems - Minneapolis, USA) and a multiplex ELISA system (BioPlex®200, Bio-Rad Laboratories, California, USA).
- **Gene expression analysis.** Frozen tissue samples will be homogenized with Trizol in RNA-free glass tubes and centrifuged at 500g for 5 minutes. Subsequently, RNA will be extracted according to the manufacturer specifications. RNA quality control will be checked by RNA electrophoreses. Finally, RNA isolation will be performed using the RNeasy MinElute Cleanup Kit (Qiagen, Germany) and DNA will be obtained by using iScript cDNA synthesis kit (Bio-Rad, USA). Target DNA will be quantified by real-time PCR using TaqMan assay according to the specifications provided by the manufacturer (Applied Biosystems, Foster City, CA, USA).

## 5. Calculations

The combination of OGTT and IVGTT will allow a comprehensive assessment of different aspects of beta cell function along with reliable measures of insulin sensitivity assessed during the euglycaemic clamp procedure [62]. Implementation of OGTT and isoglycaemic iv. glucose infusion will allow the quantification of the incretin effect based on established experimental model [63].

From OGTT data, following empirical indices of beta cell function will be derived: a) insulinogenic index (IGI) calculated as (30 min insulin – basal insulin)/(30min glucose – basal glucose); b) the early insulin response (EIR) calculated using formula (30 min insulin – basal insulin)/(30 min glucose)[64] or c) ratio of AUC for C-peptide divided by AUC for glucose (AUC_CP_/AUC_GLU_). AUCs are determined by trapezoidal method. Additionally, we intend to implement the established mathematical modelling from the OGTT to describe distinct features of beta cell function [62, 65-67]. The model describes the relationship between insulin secretion and glucose concentration coupled with a model of C-peptide kinetics. It provides not only basal insulin secretion rate and total insulin secretion during OGTT, but also three important indices of dynamic properties of beta cell function: beta cell sensitivity to glucose (glucose sensitivity), beta cell sensitivity to the rate of change of glucose (rate sensitivity) and a potentiation factor (representing relative potentiation of insulin secretion throughout the OGTT).

Based on IVGTT measurements, the incremental trapezoidal area of insulin and C-peptide during the first 10 min after glucose administration represents the first-phase insulin response or acute insulin response (AIR). AIR will be also measured as the sum of insulin and C-peptide concentrations during the first 10 min after the glucose challenge. The incremental insulin/C-peptide secretion during the last 50 min of IVGTT represents the second-phase insulin secretion. The C-peptide deconvolution will be applied to reconstruct insulin secretory rates independently of insulin clearance. It has also been proved that rate sensitivity derived from OGTT is correlated to AIR calculated from IVGTT and it is therefore a valuable marker of first phase insulin secretion [67].

The time profiles of hormones and incretins measured during OGTT and isoglycaemic i.v. glucose infusion will be expressed as AUCs. The incretin effect will be calculated as the ratio of oral to intravenous plasma insulin and C-peptide responses (as per original definition [63]). Additionally a mathematical model of beta cell function can be also employed to quantify the incretin effect on absolute insulin secretion and beta cell glucose sensitivity [3, 68].

Insulin sensitivity will be assessed as the mean glucose uptake, calculated from the glucose infusion rates during the last 60 min of euglycaemic clamp (M-value) and as the ratio of glucose infusion rates during the last 60 min of clamp and the mean steady state insulin levels during the same interval (M/I = S_I_ Index).

The disposition index (DI) as a measure of insulin secretion adjusted for insulin sensitivity will be calculated from the product of AIR and the M-value.

## 6. Statistical analysis

All data will be checked for distribution normality. In case of non-Gaussian distribution, log transformed data will be assured for further statistical evaluation.

1. Changes in beta cell function and other variables after gastric bypass surgery will be assessed by analysis of variance (ANOVA) with repeated measures. ANOVA models will include following factors:
   1. group factor as the between-subject factor (comparison between groups of bariatric patients with short- and long-standing T2DM and bariatric patients without T2DM)
   2. time factor (repeated measurements at baseline and two follow-up visits)
   3. treatment factor if appropriate - in case of repeated measurements within 1 follow-up (eg. GLP-1 profile during OGTT, basal + insulin-stimulated concentrations of adipokines and cytokines)

Time and treatment factors as the within-subject factors account for the repetition in the ANOVA model.

1. Relations between changes in beta cell function and clinical characteristics or changes in parameters of adipoinsular and enteroinsular axes will be evaluated using multivariate regression analyses.
2. For comparison of beta cell function and other variables after weight loss achieved following gastric bypass with results from BMI-matched non-bariatric controls, Mann-Whitney *U* test will be used.

Based of foregoing studies [10, 11, 19-21] a sample size of at least 15 subjects per subgroup can be safely expected to provide a sufficient statistical power to detect meaningful changes in beta cell function. Our sample size is based on feasible recruitment expectations at both sites: total number of 85 patients with an estimated drop-out rate of 10-12% during the follow-up, giving the final sample size of 75 patients – 50 patients with T2DM (among them approx. 40-50% with long-standing T2DM) and 25 patients without T2DM.

Nevertheless, based on published data [19] we have estimated mean values, variation and time-dependent covariance in individual groups for AIR (pmol/l) as a dependent variable characterizing the primary outcome. These data were used for power analysis for repeated measures ANOVA model consisting of Subject factor, Group as the between-subject factor and Time as the within-subject factor. For the statistical significance α=0.05 and power>0.8 (>80% chance to find significant relationships of factors and interaction with the dependent variable for all factors and interaction), at least 9 subjects per group should be included in the study. For the significance α=0.01 and power>0.8 for all factors and interaction, at least 13 subjects per group should be investigated. When considering power>0.95 we will need per group 13 subject for α=0.05 and 18 subjects for α=0.01. This means that 25 subjects per group will be fully sufficient for finding significant associations.

Moreover, a total number of 85 subjects will enable to perform reliable multivariate regression analyses in order to elucidate which factors, e.g. specific clinical characteristics or changes in gene expression, are most closely related to the improvement in beta cell function.

# Novelty and importance of the proposed work

For the first time, our study will systematically evaluate beta cell function, by using three different glucose stimulation tests, in relation to adipoinsular and enteroinsular axes early and long-term after gastric bypass. Moreover, our study will also address the important question, if surgery induced beta cell function improvement also occurs after longer duration of T2DM when reduction of beta cell mass is expected. The parallel performance of an OGTT and an isoglycemic iv. glucose infusion will allow to quantify the incretin effects and thus, to precisely define the role of incretin hormones in the improvement of beta cell function after bariatric surgery. Our findings are therefore expected to bring new knowledge on possible reversibility of beta cell damage in long-standing T2DM.

Our approach of repeated biopsy at 1 year follow-up and comparison with control group matched for expected one-year postoperative BMI shall enable to identify surgery-specific changes in gene expression in adipose tissue independent on weight loss.

The sample size of this double-centre study which also includes controls will enable to achieve sufficient power of the study to reliably detect significant differences between groups as well as to perform valid multivariate regression analysis.

It is worth to point out that only the combined effort of our two study centres will let us achieve such a large sample size recruited in an acceptable period of time.

The results are expected to provide important new insight into the pathophysiology of disturbed beta cell function and its possible restoration in T2DM. Bariatric operations such as the gastric bypass procedure provide a unique model of rapid changes in pathological beta cell function and the results of this study on the long run may help to develop rational approaches to overcome beta cell failure in T2DM also with conservative means, e.g. by pharmacotherapy.

# Facilities available

**Department of Endocrinology and Nuclear Medicine, Medical University of Graz, Austria**

As an integral part of Medical University of Graz and adjacent University Clinics, the Department of Endocrinology and Nuclear Medicine (Head: Prof. Thomas Pieber) includes in- and outpatient facilities, which cover complex medical care for endocrine and metabolic diseases, including treatment of severe obesity. The Outpatient Clinic provides medical consultations for treatment of all endocrine diseases including diabetes, obesity and their complications, patient education and counselling programs. The health care for patients with severe obesity proceeds in close collaboration with the Department of General Surgery of Medical University Graz (Head: Prof. Hans-Jörg Mischinger) ensuring comprehensive bariatric care.

The broad research scopes of the department span from bone and calcium metabolism and osteoporosis, over polycystic ovary syndrome, sex and thyroid hormones issues, to the pathophysiology of metabolism, diabetes mellitus and obesity and their complications. Further research focuses on the development of new research technologies in diabetes and metabolism (open-flow microperfusion, clamp techniques, tracer techniques) and on the investigation of new pharmaceutical approaches for the treatment of diabetes mellitus.

**Clinical Research Centre, Medical University of Graz, Austria**

Complex research facilities for investigations in humans are available in the Clinical Research Centre (CRC) at the Centre for Medical Research, Medical University of Graz. The presented study will be performed in the CRC, where the developed infrastructure is available to conduct the human experimental studies in accordance with GCP guidelines. The Department of Endocrinology and Nuclear Medicine has full access to all facilities of the Clinical Research Centre.

**Laboratory for Endocrinology and Metabolism, and EndoGenLab, Medical University of Graz, Austria**

On the basis of the Laboratory for Endocrinology and Metabolism (Head: Prof. Barbara Obermayer-Pietsch), founded in 1962, the Laboratory for biochemical and molecular aspects of endocrine diseases (EndoGenLab) has been certified and acknowledged as a genetic laboratory by the Austrian Ministry and by the Austrian Society for Quality Control (ISO 9008 certification, first in 2006). The qualified staff is performing analyses of biochemical as well as genetic parameters and functional endocrine tests for scientific and routine purposes. The established methods include immunochemical analysis for endocrine and metabolic parameters (ELISA, RIA, LIA), gene expression analysis via real-time PCR or SNP genotyping. The annual output is about 250.000 tests in all fields of hormonal and metabolic analyses.

**Interdisciplinary Obesity Center, St.Gallen, Switzerland**

The Interdisciplinary Obesity Center St. Gallen offers the full range of up-to-date obesity therapy spanning from psychotherapy to bariatric surgery. Every year about 400 new patients report to the Interdiscipilnäry Obesity Center of whom approximately 160 undergo an bariatric operation. Currently, about 1000 operated patients are included in a multiprofessional structured follow-up programme. On background, the applicant from St. Gallen has an extented clinical experience in the management of obese and especially bariatric patients.

**Research laboratory of the Interdisciplinary Obesity Center, St. Gallen, Switzerland**

The obesity research laboratory (Head: Prof. Bernd Schultes) is equipped with a wide range of technical facilities to perform comprehensive metabolic and biopsychological characterisation studies in bariatric and non-bariatric patients. The obesity research laboratory is joint with the Medical Research Center (Head: Prof. Burghard Ludewig) as well as with the Clinical Trial Unit (funded by the Swiss National Foundation; Head: Prof. Christoph Driesen) of the Kantonsspital St. Gallen, and thus surrounded by other high quality research facilities. The main focus of the obesity research laboratory is to performed human experimental studies in the field of obesity and diabetes in accordance with GCP guidelines.

# Collaborative arrangements

**The Biobank of the Medical University of Graz, Austria** is specifically designed to support the needs of systems biology approaches to human diseases, drug discovery and public health. It is a research core facility of Medical University of Graz, which builds on different collections of human biomaterial (Blood, DNA, RNA, FFPE, Cryotissue, placenta, etc.) and represents a unique combination of Population-based biobank and Disease-based biobank. It comprehends one of the world's largest most consistent collection of diseased human tissues of the Institute of Pathology (healthy and diseased tissue, containing 3.3 million tissue samples from more than 880,000 patients) and one of the European one-of-a-kind oncology serum bank (400000 samples from app. 100000 patients in follow-up). The biobank will be further developed into a biological resource centre putting specific emphasis on standardization, international networking and the ethical, legal and societal issues (ELSI) of biobanking.

Based on systematic experimental evidence, best practice guidelines for sample-preservation and pre-analytical processing for intervention studies, sample collection standards will be developed specifically for the planned study, addressing the needs of collaboration of two centres.

The letter of confirmation is attached as Appendix 5.

# References

[1] Hamman RF, Wing RR, Edelstein SL, et al. (2006) Effect of weight loss with lifestyle intervention on risk of diabetes. Diabetes Care 29: 2102-2107

[2] Ferrannini E, Camastra S, Gastaldelli A, et al. (2004) beta-cell function in obesity: effects of weight loss. Diabetes 53 Suppl 3: S26-33

[3] Muscelli E, Mari A, Casolaro A, et al. (2008) Separate impact of obesity and glucose tolerance on the incretin effect in normal subjects and type 2 diabetic patients. Diabetes 57: 1340-1348

[4] Cummings DE, Flum DR (2008) Gastrointestinal surgery as a treatment for diabetes. JAMA 299: 341-343

[5] Buchwald H, Avidor Y, Braunwald E, et al. (2004) Bariatric surgery: a systematic review and meta-analysis. JAMA 292: 1724-1737

[6] Smith BR, Hinojosa MW, Reavis KM, Nguyen NT (2008) Remission of diabetes after laparoscopic gastric bypass. Am Surg 74: 948-952

[7] Scopinaro N, Papadia F, Camerini G, Marinari G, Civalleri D, Gian Franco A (2008) A comparison of a personal series of biliopancreatic diversion and literature data on gastric bypass help to explain the mechanisms of resolution of type 2 diabetes by the two operations. Obes Surg 18: 1035-1038

[8] Mottin CC, Vontobel Padoin A, Schroer CE, Barancelli FT, Glock L, Repetto G (2008) Behavior of type 2 diabetes mellitus in morbid obese patients submitted to gastric bypass. Obes Surg 18: 179-181

[9] Schauer PR, Burguera B, Ikramuddin S, et al. (2003) Effect of laparoscopic Roux-en Y gastric bypass on type 2 diabetes mellitus. Ann Surg 238: 467-484; discussion 484-465

[10] Guidone C, Manco M, Valera-Mora E, et al. (2006) Mechanisms of recovery from type 2 diabetes after malabsorptive bariatric surgery. Diabetes 55: 2025-2031

[11] Mari A, Manco M, Guidone C, et al. (2006) Restoration of normal glucose tolerance in severely obese patients after bilio-pancreatic diversion: role of insulin sensitivity and beta cell function. Diabetologia 49: 2136-2143

[12] Bikman BT, Zheng D, Pories WJ, et al. (2008) Mechanism for improved insulin sensitivity after gastric bypass surgery. J Clin Endocrinol Metab 93: 4656-4663

[13] Rosa G, Mingrone G, Manco M, et al. (2007) Molecular mechanisms of diabetes reversibility after bariatric surgery. Int J Obes (Lond) 31: 1429-1436

[14] Greco AV, Mingrone G, Giancaterini A, et al. (2002) Insulin resistance in morbid obesity: reversal with intramyocellular fat depletion. Diabetes 51: 144-151

[15] Salinari S, Bertuzzi A, Iaconelli A, Manco M, Mingrone G (2008) Twenty-four hour insulin secretion and beta cell NEFA oxidation in type 2 diabetic, morbidly obese patients before and after bariatric surgery. Diabetologia 51: 1276-1284

[16] Camastra S, Manco M, Mari A, et al. (2007) Beta-cell function in severely obese type 2 diabetic patients: long-term effects of bariatric surgery. Diabetes Care 30: 1002-1004

[17] Rubino F (2008) Is type 2 diabetes an operable intestinal disease? A provocative yet reasonable hypothesis. Diabetes Care 31 Suppl 2: S290-296

[18] Bose M, Olivan B, Teixeira J, Pi-Sunyer FX, Laferrere B (2009) Do Incretins Play a Role in the Remission of Type 2 Diabetes after Gastric Bypass Surgery: What are the Evidence? Obes Surg 19: 217-229

[19] Polyzogopoulou EV, Kalfarentzos F, Vagenakis AG, Alexandrides TK (2003) Restoration of euglycemia and normal acute insulin response to glucose in obese subjects with type 2 diabetes following bariatric surgery. Diabetes 52: 1098-1103

[20] Briatore L, Salani B, Andraghetti G, et al. (2008) Restoration of acute insulin response in T2DM subjects 1 month after biliopancreatic diversion. Obesity (Silver Spring) 16: 77-81

[21] Salinari S, Bertuzzi A, Asnaghi S, Guidone C, Manco M, Mingrone G (2009) First-phase insulin secretion restoration and differential response to glucose load depending on the route of administration in type 2 diabetic subjects after bariatric surgery. Diabetes Care 32: 375-380

[22] Rodieux F, Giusti V, D'Alessio DA, Suter M, Tappy L (2008) Effects of gastric bypass and gastric banding on glucose kinetics and gut hormone release. Obesity (Silver Spring) 16: 298-305

[23] Laferrere B, Teixeira J, McGinty J, et al. (2008) Effect of weight loss by gastric bypass surgery versus hypocaloric diet on glucose and incretin levels in patients with type 2 diabetes. J Clin Endocrinol Metab 93: 2479-2485

[24] Buchwald H, Williams SE (2004) Bariatric surgery worldwide 2003. Obes Surg 14: 1157-1164

[25] Gumbs AA, Modlin IM, Ballantyne GH (2005) Changes in insulin resistance following bariatric surgery: role of caloric restriction and weight loss. Obes Surg 15: 462-473

[26] Rosen ED, Spiegelman BM (2006) Adipocytes as regulators of energy balance and glucose homeostasis. Nature 444: 847-853

[27] Zhao YF, Feng DD, Chen C (2006) Contribution of adipocyte-derived factors to beta-cell dysfunction in diabetes. Int J Biochem Cell Biol 38: 804-819

[28] Zhao YF, Chen C (2007) Regulation of pancreatic beta-cell function by adipocytes. Sheng Li Xue Bao 59: 247-252

[29] Donath MY, Schumann DM, Faulenbach M, Ellingsgaard H, Perren A, Ehses JA (2008) Islet inflammation in type 2 diabetes: from metabolic stress to therapy. Diabetes Care 31 Suppl 2: S161-164

[30] Swarbrick MM, Stanhope KL, Austrheim-Smith IT, et al. (2008) Longitudinal changes in pancreatic and adipocyte hormones following Roux-en-Y gastric bypass surgery. Diabetologia 51: 1901-1911

[31] Geloneze B, Tambascia MA, Pareja JC, Repetto EM, Magna LA, Pereira SG (2001) Serum leptin levels after bariatric surgery across a range of glucose tolerance from normal to diabetes. Obes Surg 11: 693-698

[32] Ballantyne GH, Gumbs A, Modlin IM (2005) Changes in insulin resistance following bariatric surgery and the adipoinsular axis: role of the adipocytokines, leptin, adiponectin and resistin. Obes Surg 15: 692-699

[33] Guldstrand M, Ahren B, Adamson U (2003) Improved beta-cell function after standardized weight reduction in severely obese subjects. Am J Physiol Endocrinol Metab 284: E557-565

[34] Swarbrick MM, Austrheim-Smith IT, Stanhope KL, et al. (2006) Circulating concentrations of high-molecular-weight adiponectin are increased following Roux-en-Y gastric bypass surgery. Diabetologia 49: 2552-2558

[35] Coughlin CC, Finck BN, Eagon JC, et al. (2007) Effect of marked weight loss on adiponectin gene expression and plasma concentrations. Obesity (Silver Spring) 15: 640-645

[36] Engl J, Bobbert T, Ciardi C, et al. (2007) Effects of pronounced weight loss on adiponectin oligomer composition and metabolic parameters. Obesity (Silver Spring) 15: 1172-1178

[37] Vilarrasa N, Vendrell J, Sanchez-Santos R, et al. (2007) Effect of weight loss induced by gastric bypass on proinflammatory interleukin-18, soluble tumour necrosis factor-alpha receptors, C-reactive protein and adiponectin in morbidly obese patients. Clin Endocrinol (Oxf) 67: 679-686

[38] Catalan V, Gomez-Ambrosi J, Ramirez B, et al. (2007) Proinflammatory cytokines in obesity: impact of type 2 diabetes mellitus and gastric bypass. Obes Surg 17: 1464-1474

[39] Kopp HP, Kopp CW, Festa A, et al. (2003) Impact of weight loss on inflammatory proteins and their association with the insulin resistance syndrome in morbidly obese patients. Arterioscler Thromb Vasc Biol 23: 1042-1047

[40] Whitson BA, Leslie DB, Kellogg TA, et al. (2007) Adipokine response in diabetics and nondiabetics following the Roux-en-Y gastric bypass: a preliminary study. J Surg Res 142: 295-300

[41] Ernst B, Thurnheer M, Wilms B, Schultes B (2009) Differential changes in dietary habits after gastric bypass versus gastric banding operations. Obes Surg 19: 274-280

[42] Ernst B, Thurnheer M, Schmid SM, Wilms B, Schultes B (2009) Seasonal variation in the deficiency of 25-hydroxyvitamin d(3) in mildly to extremely obese subjects. Obes Surg 19: 180-183

[43] Ernst B, Thurnheer M, Schmid SM, Schultes B (2009) Evidence for the necessity to systematically assess micronutrient status prior to bariatric surgery. Obes Surg 19: 66-73

[44] Schmid SM, Jauch-Chara K, Hallschmid M, et al. (2008) Lactate overrides central nervous but not beta-cell glucose sensing in humans. Metabolism 57: 1733-1739

[45] Schmid SM, Hallschmid M, Jauch-Chara K, Bandorf N, Born J, Schultes B (2007) Sleep loss alters basal metabolic hormone secretion and modulates the dynamic counterregulatory response to hypoglycemia. J Clin Endocrinol Metab 92: 3044-3051

[46] Jauch-Chara K, Hallschmid M, Gais S, et al. (2007) Hypoglycemia during sleep impairs consolidation of declarative memory in type 1 diabetic and healthy humans. Diabetes Care 30: 2040-2045

[47] Jauch-Chara K, Hallschmid M, Gais S, et al. (2007) Awakening and counterregulatory response to hypoglycemia during early and late sleep. Diabetes 56: 1938-1942

[48] Schultes B, Jauch-Chara K, Gais S, et al. (2007) Defective awakening response to nocturnal hypoglycemia in patients with type 1 diabetes mellitus. PLoS Med 4: e69

[49] Schultes B, Peters A, Hallschmid M, et al. (2005) Modulation of food intake by glucose in patients with type 2 diabetes. Diabetes Care 28: 2884-2889

[50] Pachler C, Ikeoka D, Plank J, et al. (2007) Subcutaneous adipose tissue exerts proinflammatory cytokines after minimal trauma in humans. Am J Physiol Endocrinol Metab 293: E690-696

[51] Weinhandl H, Pachler C, Mader JK, et al. (2007) Physiological hyperinsulinemia has no detectable effect on access of macromolecules to insulin-sensitive tissues in healthy humans. Diabetes 56: 2213-2217

[52] Ikeoka D, Pachler C, Korsatko S, et al. (2007) Local Stimulated Secretion of Inflammatory Cytokines from Adipose Tissue is Reduced in Septic Patients in Comparison to Healthy Controls. In: 15th Annual Meeting of the International Cytokine Society. Cytokines 2007, San Francisco, USA

[53] Ikeoka D, Pachler C, Mader J, et al. (2007) Non-Esterified Fatty Acids Induce Insulin Resistance and Promote a Systemic increase of Macrophage Chemoattractant Protein-1 and Plasminogen Activator Inhibitor-1 in Humans. In: 15th Annual Meeting of the International Cytokine Society. Cytokines 2007, San Francisco, USA

[54] van Meurs JB, Trikalinos TA, Ralston SH, et al. (2008) Large-scale analysis of association between LRP5 and LRP6 variants and osteoporosis. JAMA 299: 1277-1290

[55] Marz W, Seelhorst U, Wellnitz B, et al. (2007) Alanine to serine polymorphism at position 986 of the calcium-sensing receptor associated with coronary heart disease, myocardial infarction, all-cause, and cardiovascular mortality. J Clin Endocrinol Metab 92: 2363-2369

[56] Herberth J, Fahrleitner-Pammer A, Obermayer-Pietsch B, et al. (2006) Changes in total parathyroid hormone (PTH), PTH-(1-84) and large C-PTH fragments in different stages of chronic kidney disease. Clin Nephrol 65: 328-334

[57] Fahrleitner-Pammer A, Obernosterer A, Pilger E, et al. (2005) Hypovitaminosis D, impaired bone turnover and low bone mass are common in patients with peripheral arterial disease. Osteoporos Int 16: 319-324

[58] Obermayer-Pietsch BM, Bonelli CM, Walter DE, et al. (2004) Genetic predisposition for adult lactose intolerance and relation to diet, bone density, and bone fractures. J Bone Miner Res 19: 42-47

[59] Obermayer-Pietsch BM, Lange U, Tauber G, et al. (2003) Vitamin D receptor initiation codon polymorphism, bone density and inflammatory activity of patients with ankylosing spondylitis. Osteoporos Int 14: 995-1000

[60] Obermayer-Pietsch BM, Fruhauf GE, Lipp RW, Sendlhofer G, Pieber TR (2001) Dissociation of leptin and body weight in hyperthyroid patients after radioiodine treatment. Int J Obes Relat Metab Disord 25: 115-120

[61] Tripathy D, Wessman Y, Gullstrom M, Tuomi T, Groop L (2003) Importance of obtaining independent measures of insulin secretion and insulin sensitivity during the same test: results with the Botnia clamp. Diabetes Care 26: 1395-1401

[62] Ferrannini E, Mari A (2004) Beta cell function and its relation to insulin action in humans: a critical appraisal. Diabetologia 47: 943-956

[63] Nauck MA, Homberger E, Siegel EG, et al. (1986) Incretin effects of increasing glucose loads in man calculated from venous insulin and C-peptide responses. J Clin Endocrinol Metab 63: 492-498

[64] Wareham NJ, Phillips DI, Byrne CD, Hales CN (1995) The 30 minute insulin incremental response in an oral glucose tolerance test as a measure of insulin secretion. Diabet Med 12: 931

[65] Mari A, Schmitz O, Gastaldelli A, Oestergaard T, Nyholm B, Ferrannini E (2002) Meal and oral glucose tests for assessment of beta -cell function: modeling analysis in normal subjects. Am J Physiol Endocrinol Metab 283: E1159-1166

[66] Mari A, Tura A, Gastaldelli A, Ferrannini E (2002) Assessing insulin secretion by modeling in multiple-meal tests: role of potentiation. Diabetes 51 Suppl 1: S221-226

[67] Mari A, Ferrannini E (2008) Beta-cell function assessment from modelling of oral tests: an effective approach. Diabetes Obes Metab 10 Suppl 4: 77-87

[68] Muscelli E, Mari A, Natali A, et al. (2006) Impact of incretin hormones on beta-cell function in subjects with normal or impaired glucose tolerance. Am J Physiol Endocrinol Metab 291: E1144-1150

# Abstract pages from all other sources of support

See Appendix 1-4.
