# Supplementary material for: An Untargeted Metabolomics Approach to Characterize Short-Term and Long-Term Metabolic Changes after Bariatric Surgery
Source: PLoS One. 2016 Sep 1;11(9):e0161425. doi: 10.1371/journal.pone.0161425 (PMC5008721; doi:10.1371/journal.pone.0161425)
Supplement: S3 Text — (PPTX) [file pone.0161425.s011.pptx]

## Slide 1
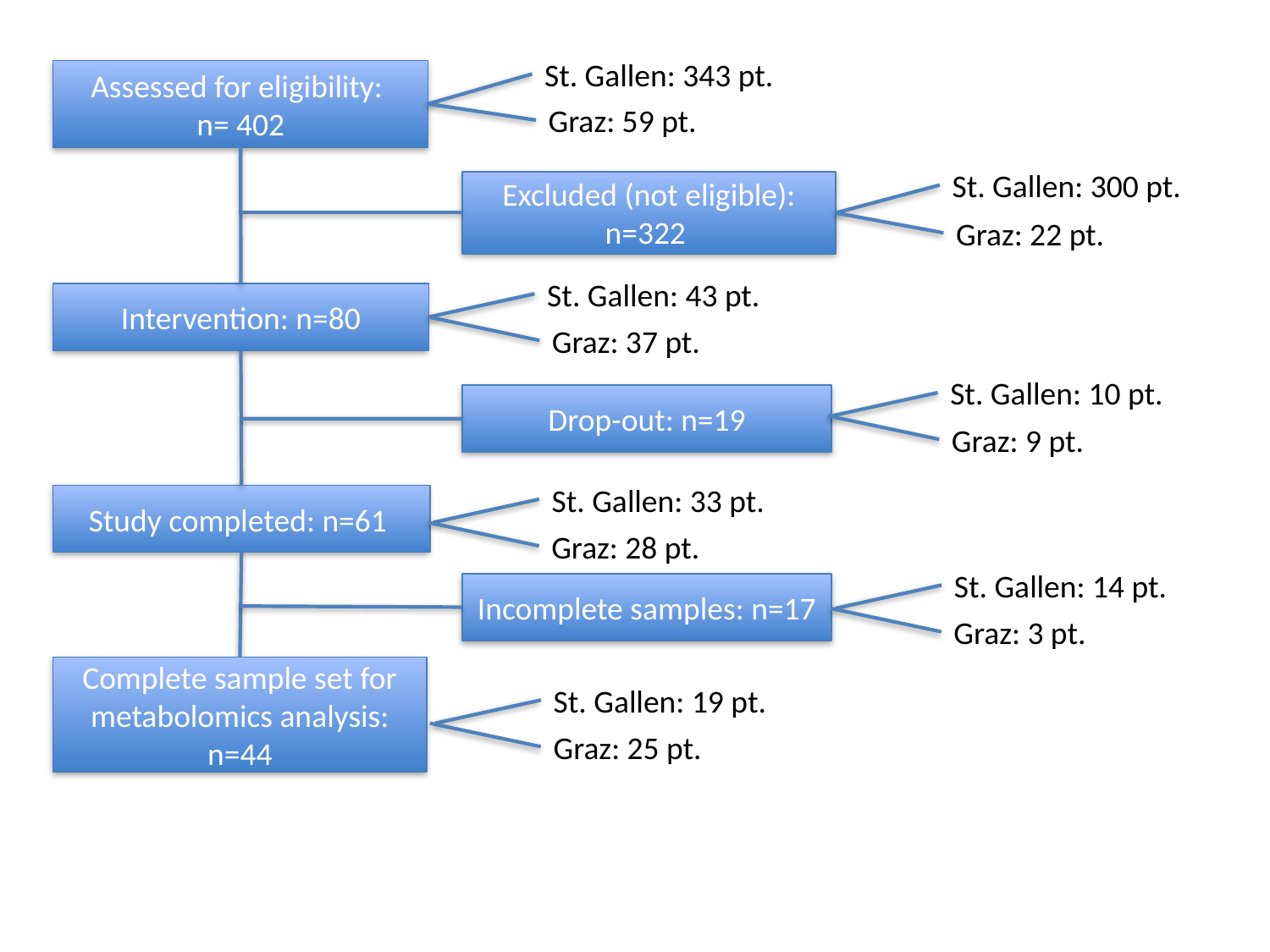

St. Gallen: 343 pt.
Assessed for eligibility:
n= 402
Graz: 59 pt.
St. Gallen: 300 pt.
Excluded (not eligible): n=322
Graz: 22 pt.
St. Gallen: 43 pt.
Intervention: n=80
Graz: 37 pt.
St. Gallen: 10 pt.
Drop-out: n=19
Graz: 9 pt.
St. Gallen: 33 pt.
Study completed: n=61
Graz: 28 pt.
St. Gallen: 14 pt.
Incomplete samples: n=17
Graz: 3 pt.
Complete sample set for metabolomics analysis: n=44
St. Gallen: 19 pt.
Graz: 25 pt.
